# Epigenetic Mechanisms in Heart Diseases

**DOI:** 10.31083/RCM38696

**Published:** 2025-07-30

**Authors:** Mustafa Yildiz

**Affiliations:** ^1^Medical Faculty, Department of Biophysics, Trakya University, 22030 Edirne, Türkiye

**Keywords:** epigenetics, heart diseases, microRNAs, HDACs, SIRTs, DNMTs, TETs, drugs, non-coding RNAs

## Abstract

Heart diseases (HDs) continue to be among the major diseases that adversely affect human health worldwide, with complex interactions between genetic, environmental, and biochemical factors contributing to their progression. These include coronary heart disease, hypertension, heart failure, vascular calcification, etc. Cardiovascular diseases have been extensively studied in the Framingham Heart Study since 1948, spanning three generations over the past 70 years, and are highly correlated with various factors, including biochemical, environmental, behavioral, and genetic factors. In recent years, epigenetic mechanisms have emerged as crucial regulators of cardiovascular pathology, influencing gene expression without altering the underlying DNA sequence. Moreover, early detection and diagnosis of heart diseases are crucial for improving treatment and prognosis. Recent studies on heart disease have found that the expression of potential candidate genes related to the disease is associated with epigenetic mechanisms. Indeed, abnormal methylation states have been detected in candidate genes that can serve as biomarkers to assess the progression of heart disease. Recent advances in next-generation sequencing techniques have contributed significantly to our understanding of heart diseases, including the role of DNA methylation, adenosine triphosphate (ATP)-dependent chromatin conformation and remodeling, post-translational modifications of histones and non-coding RNAs. Lastly, this review examines the latest discoveries in the epigenetic regulation of heart diseases, highlighting the roles of DNA methyltransferases (DNMTs), histone deacetylases (HDACs), sirtuins (SIRTs), and ten-eleven translocation proteins (TETs). Additionally, this review highlights preclinical therapeutic strategies targeting epigenetic modifiers, offering new avenues for precision medicine in cardiology. Understanding these epigenetic pathways is crucial for developing novel biomarkers and epigenetic-based therapies that aim to reverse maladaptive cardiac remodeling and enhance clinical outcomes.

## 1. Introduction

Data from the World Health Organization (WHO) for 194 countries indicate that noncommunicable diseases (NCDs), with a particular emphasis on heart diseases, are responsible for nearly 75% of deaths globally [1, 2]. In 2019, cardiovascular diseases (CVDs) caused 17.9 million deaths worldwide, which is 32% of the total global mortality, with 85% of these deaths linked to heart disease and stroke [1]. Approximately 80–90% of the mass of cardiac cells consists of cardiomyocytes, yet these cells represent only about 30–50% of the different cell types that exist in the heart. The prevalence of heart failure (HF) is a substantial public health challenge, currently impacting close to 6 million people in the USA, and it is projected to escalate to 8.5 million by 2030 [3, 4].

Genetic modifications have been acknowledged for an extended period as significant factors in the development of various diseases, encompassing mutations, deletions, and chromosomal rearrangements. Epigenetic regulatory mechanisms are characterized by their capacity to adjust gene expression without modifying the underlying DNA sequences. Epigenetic histone modifications exhibit a more nuanced regulatory function compared to DNA methylation, where the specific methylation locations within the genome significantly influence the roles of histones. Moreover, histones operate at multiple levels, including genomic levels via DNA methylation, as well as at the nucleosomal and chromatin levels through modifications and chromatin remodeling complexes. Moreover, small non-coding RNAs, known as microRNAs (miRs), contribute to an additional regulatory layer at the post-transcriptional stage. Most methylated cytosines are situated adjacent to guanine bases; a considerable number of active genes display a grouping of these methylated cytosines around their transcription promoter regions [5]. Cytosine-phosphate-guanine (CpG) islands, which are clusters of CpG dinucleotides, are primarily characterized by their unmethylated state. Conversely, CpG sites that are positioned between genes or within repetitive DNA sequences are typically methylated. Notably, not all CpG dinucleotides in normal mammalian cells are subject to methylation; indeed, those located in the promoter regions of CpG islands are often protected from such modifications, while CpG sites in both coding and non-coding regions of genes are usually methylated [6]. The observation led to the division of promoters into two primary categories based on their CpG density: low CpG density (LCG) and high CpG density promoters. LCG promoters represent 28% of the promoters identified in the human genome. Meanwhile, genomic areas with high CpG density are generally hypomethylated, whereas those with low CpG density are typically hypermethylated [7].

The creation of the epigenetic code occurs without altering the DNA sequence; nevertheless, some portions of the DNA may cease to hold relevance. The primary types of epigenetic modifications are those affecting histone proteins and the methylation of DNA. It is also significant to mention that epigenetic modifications can be inherited through direct or indirect pathways. Both DNA methylation and histone code modifications are acknowledged as direct mechanisms of gene regulation. In the case of DNA methylation, this process typically consists of adding a methyl group to cytosine residues within CpG islands, which ultimately leads to gene silencing. The modifications that occur after the translation of histones, known as post-translational modifications, encompass various processes, including acetylation, methylation, phosphorylation, ubiquitylation, and SUMOylation, which alter chromatin structure and accessibility, thereby influencing transcriptional activity. To exemplify, there are mother mice that frequently lick their young, while others do so less consistently. This licking behavior is known to induce demethylation in genes that are critical for managing stress responses. Therefore, offspring that are not licked and face neglect are more prone to becoming adults who experience significant stress [8]. The presence of impaired fetal growth and maternal health issues, including hypercholesterolemia, could predispose individuals to early-onset CVD through changes in gene expression driven by epigenetic factors [9]. Non-coding RNAs (ncRNAs), which encompass microRNAs and long non-coding RNAs, serve as important regulators of gene expression through indirect mechanisms. These ncRNAs do not directly alter the structure of DNA or histones; instead, these RNAs modulate the functions of transcription factors and chromatin remodeling complexes, as well as interact with other elements in the epigenetic landscape to shape gene expression profiles. Agouti mice possess a segment of repetitive DNA adjacent to their eponymous gene, which, when in an unmethylated state, perpetually activates the gene. This activation results in a yellow coat coloration, obesity, type 2 diabetes, and a heightened susceptibility to cancer. The process of methylating this DNA caused the agouti gene to be silenced, which resulted in the development of mice that were darker, slimmer, and healthier [10]. Geneticists research genes, but for epigeneticists, the groundwork has recently been laid for an obvious concept of “epigene”. Moreover, last year, epigenetics was the subject of more than 10,000 papers and numerous scientific meetings. Epigenetic regulations can turn genes on and off, as well as determine which proteins are transcribed. These regulatory mechanisms involve DNA methylation, histone modification, ncRNAs, and differential RNA splicing. In 1983, cancer became the first human disease identified as being associated with epigenetic alterations [11]. DNA methylation is the most extensively studied and understood mechanism within the realm of epigenetics. This enzymatic change occurs when cytosines are converted into 5-methylcytosines. The methylation of the fifth carbon on the cytosine base in the cytosine–guanine-rich promoter region subsequently restricts access for the transcriptional machinery. Alternatively, the removal of methyl groups from these 5-methylcytosine residues could render previously inaccessible sites accessible to regulatory elements. Modifications in the DNA sequence may lead to alterations in the three-dimensional form of the DNA molecule, which could either obstruct or enable the entry of other molecules into the DNA structure. The regulation of cell death mechanisms, such as necroptosis, pyroptosis, ferroptosis, and cuproptosis, is fundamentally influenced by epigenetic modifications, which are linked to heart disease. These regulated cell death pathways are fine-tuned by DNA methylation, histone modifications, and ncRNAs [12].

Histone deacetylases (HDACs) can affect post-translational modifications, such as ubiquitination and methylation, while also influencing gene transcription by enhancing the interaction between DNA and histones.

**Fig. 1.**
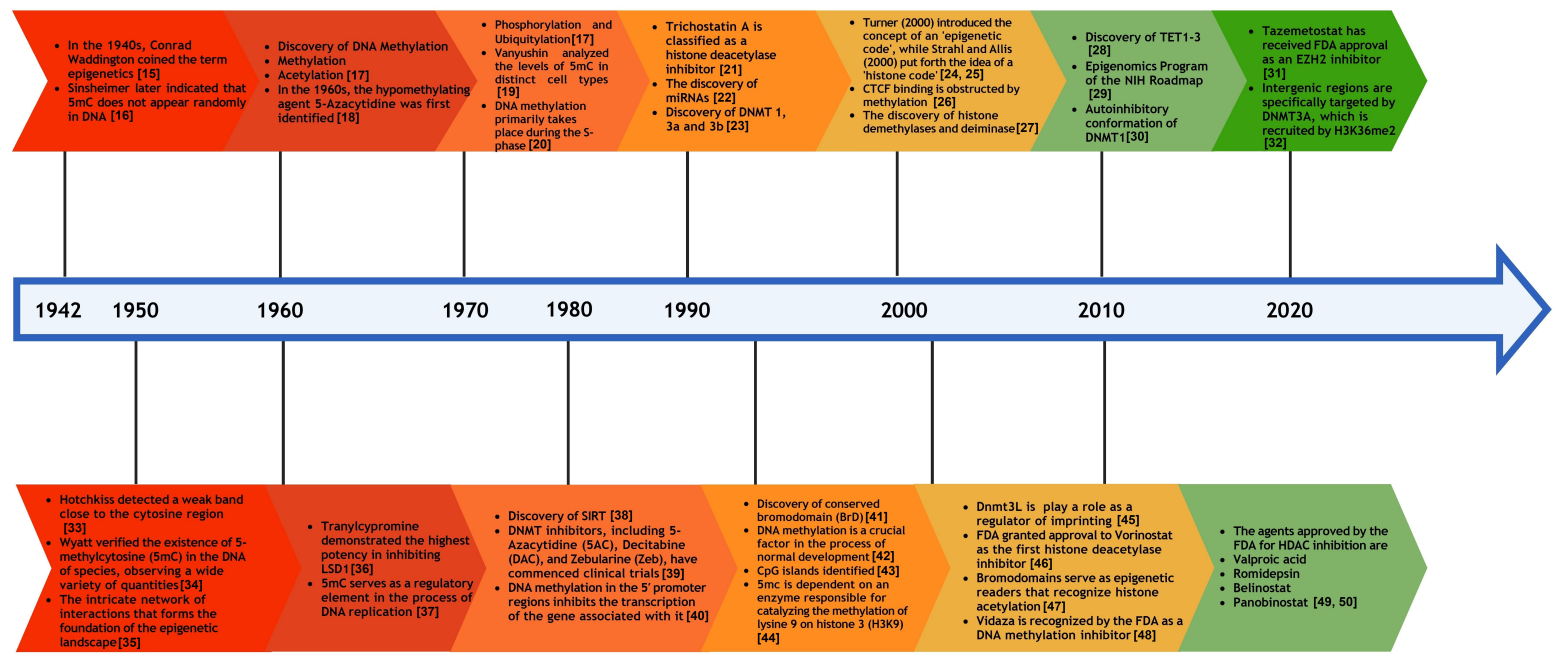
**Historical timeline of epigenetics and the development of epigenetic drugs**. 5mc, 5-methylcytosine; CTCF, CCCTC-binding factor; NIH, National Institutes of Health; FDA, Food and Drug Administration; EZH2, Enhancer of Zeste Homolog 2; LSD1, Lysine-Specific Demethylase 1; TET, ten-eleven translocation; DNMT, DNA methyltransferase; HDAC, histone deacetylase.

The process of histone acetylation is linked to the alteration in chromatin structure in transcriptionally active regions. This modification involves the addition of acetyl groups (COCH_3_) to the lysine residues located in the N-terminal tails of histones, a reaction catalyzed by histone acetyltransferases (HATs). The impact of histone modification functions is determined not solely by the sites of these modifications, including the specific amino acid residues and genomic regions, but also by the type and extent of modifications occurring on the histones. It has been suggested that epigenetic changes may help clarify the missing elements of inheritance in the sequence variations of complex diseases, including atherosclerosis, metabolic syndrome, hypertension, and diabetes, which genetic studies have yet to explain fully [13]. Thus, epigenetic alterations play a central role in the expression and, consequently, in the pathogenesis and progression of HDs by influencing the expression of genes involved in cell survival and death. Recent developments have helped open new avenues for targeting these pathways in the design of novel therapeutic strategies [14]. Fig. 1 (Ref. [15, 16, 17, 18, 19, 20, 21, 22, 23, 24, 25, 26, 27, 28, 29, 30, 31, 32, 33, 34, 35, 36, 37, 38, 39, 40, 41, 42, 43, 44, 45, 46, 47, 48, 49, 50]) presents a historical timeline of the epigenetic processes and drug development to provide a more thorough understanding of the historical discoveries and research efforts in the field of epigenetics, along with the advancement of epigenetic drug therapies.

## 2. Epigenetic Factors

### 2.1 DNA Methyltransferase Family

**Fig. 2.**
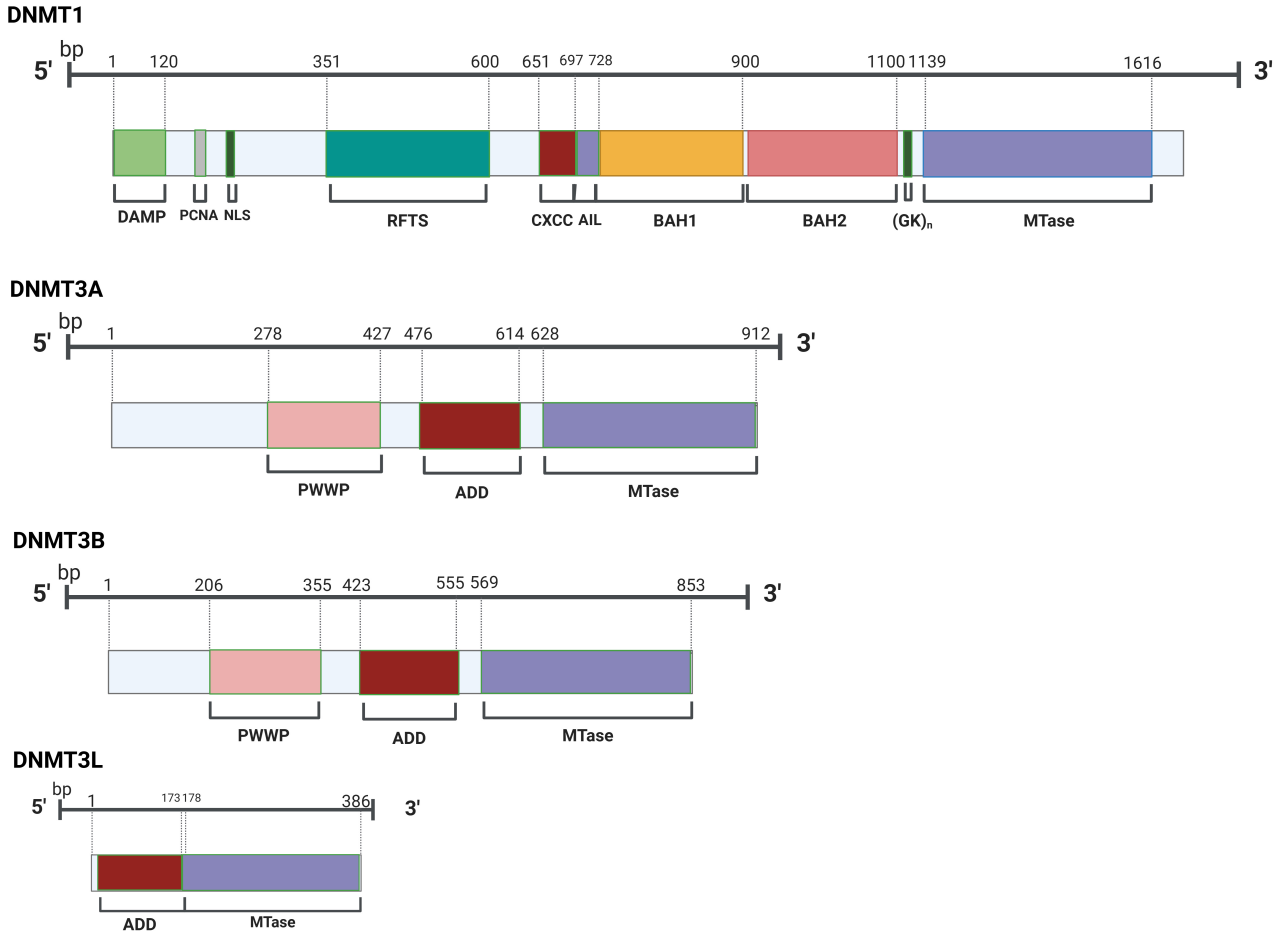
**Members of the DNMT family possess a common catalytic domain in their structural composition**. DNMT, DNA methyltransferase; DAMP, DNMT1-Associated Maintenance Protein; PCNA, Proliferating Cell Nuclear Antigen; NLS, Nuclear Localization Signal; RFTS, Replication Foci Targeting Sequence; CXXC, Cysteine-X-X-Cysteine; AIL, Autoinhibitory Linker; BAH, Bromo-Adjacent Homology; (GK)_n_, Glycine Lysine Repeats; PWWP, Pro-Trp-Trp-Pro; ADD, ATRX-DNMT3-DNMT3L; MTase, Methyltransferase.

In mammals, DNA methyltransferases comprise three members that are categorized into two families, which are distinct both structurally and functionally (Fig. 2). DNA methylation patterns are mainly mediated by DNA methyltransferase 1 (DNMT1), which is involved in replication-dependent maintenance and repair mechanisms. A protein called DNA methyltransferase 1 is responsible for copying the original DNA methylation pattern to newly formed strands. Once formed, 5-mC is considered a stable epigenetic marker, as it can be transmitted to replicating cells through DNA replication [51].

DNMT1 is a key player in HF and cardiomyopathy, with its expression elevated in response to pathological stress. The absence of DNMT1 in the myocardium confers a protective effect against cardiac damage, altering gene expression and DNA methylation patterns, and highlighting the potential impact of epigenetic regulation in heart disease [52]. DNA methylation is mediated by DNA methyltransferases 3A (DNMT3A) and 3B (DNMT3B), referred to as *de novo* DNA methyltransferases during germ cell development and early embryogenesis. DNMT3A and DNMT3B initiate the establishment of the CpG methylation pattern *de novo*, whereas DNMT1 is involved in sustaining this pattern during replication and repair of chromosomes. DNMT3L serves as a cofactor for the enzymes DNMT3A and DNMT3B. DNMT3A and DNMT3B also contain a regulatory factor, Dnmt3-like protein (DNMR3L).

### 2.2 Ten-Eleven Translocation Proteins

**Fig. 3.**
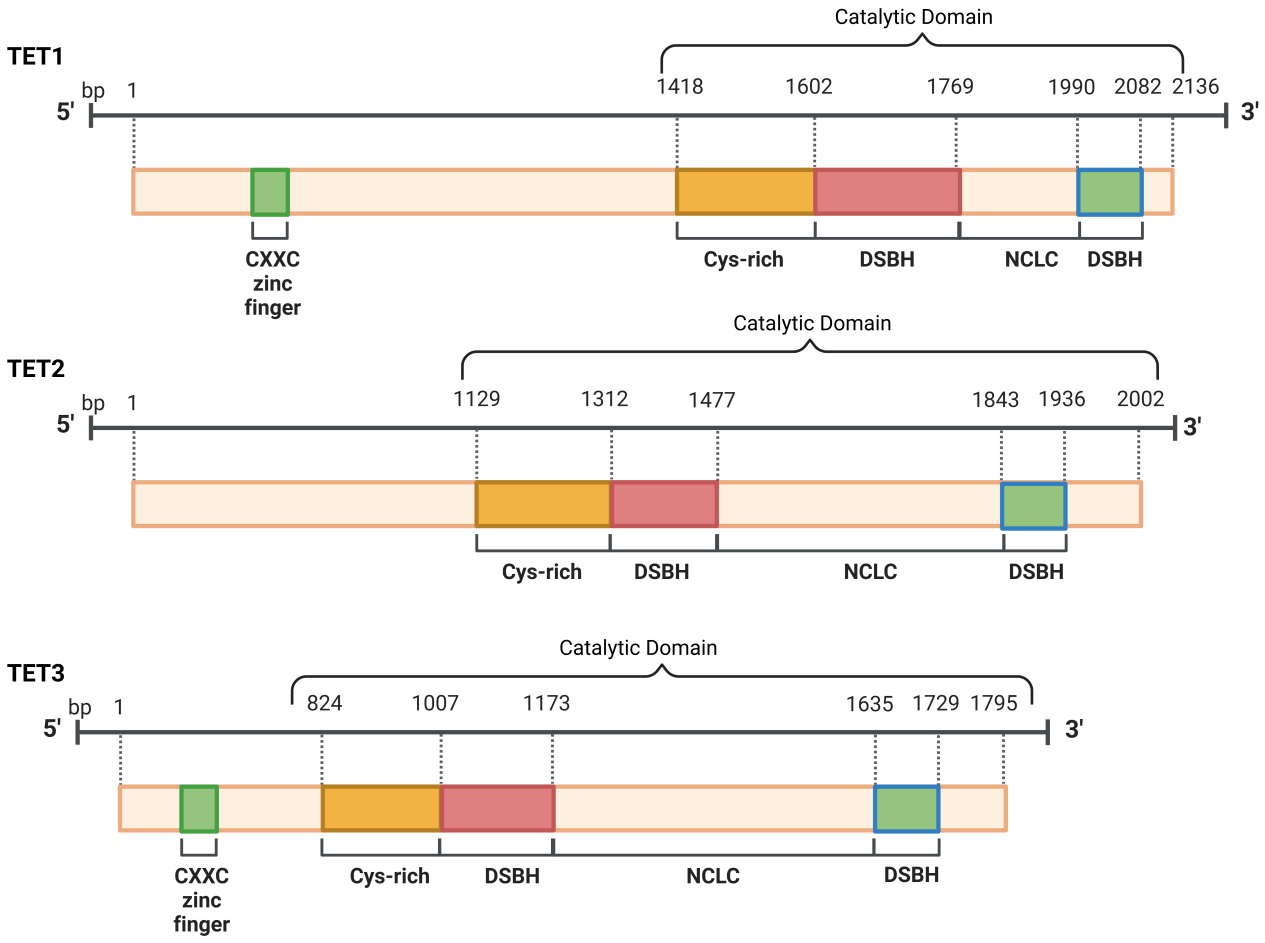
**The structural composition of the TETs is defined by the presence of one core catalytic domain at the C-terminal**. The CXXC domain is located at the N-terminal of TET1 and TET3, which facilitates direct DNA binding, whereas TET2 does not possess this domain. TET, ten-eleven translocation; CXXC, Cysteine-X-X-Cysteine; DSBH, Double-Stranded β-Helix; NCLC, N-terminal Cysteine-rich.

It has been discovered that ten-eleven translocation proteins, which exhibit similar activity on both DNA and RNA, are capable of converting RNA 5-methylcytosine (5-mC) into 5-hydroxymethylcytosine (5-hmC), thereby enhancing the translation of RNA molecules. “Eraser” proteins, called TETs (Fig. 2), bind to specifically labeled methyl groups and detach these groups from the DNA. The process of DNA demethylation is divided into two classifications: passive and active. Passive mechanisms are characterized by a failure in the repair processes that maintain DNA methylation patterns throughout replication, resulting in the dilution of hemimethylated CpGs in subsequent cycles of DNA replication (Fig. 3) [53].

Ten-eleven translocation 1 (TET1) facilitates the conversion of 5-mC into 5-hmC, which was initially identified in 2003. Subsequently, two other TET family members, TET2 and TET3, were characterized shortly after this initial study. Methyl groups that need to be removed during active demethylation are labeled with oxygen atoms. TET enzymes oxidize methylcytosines and mediate DNA demethylation. The TET protein family consists of three members: TET1, TET2, and TET3 [51].

### 2.3 Histone Deacetylases and Sirtuins Family

**Fig. 4.**
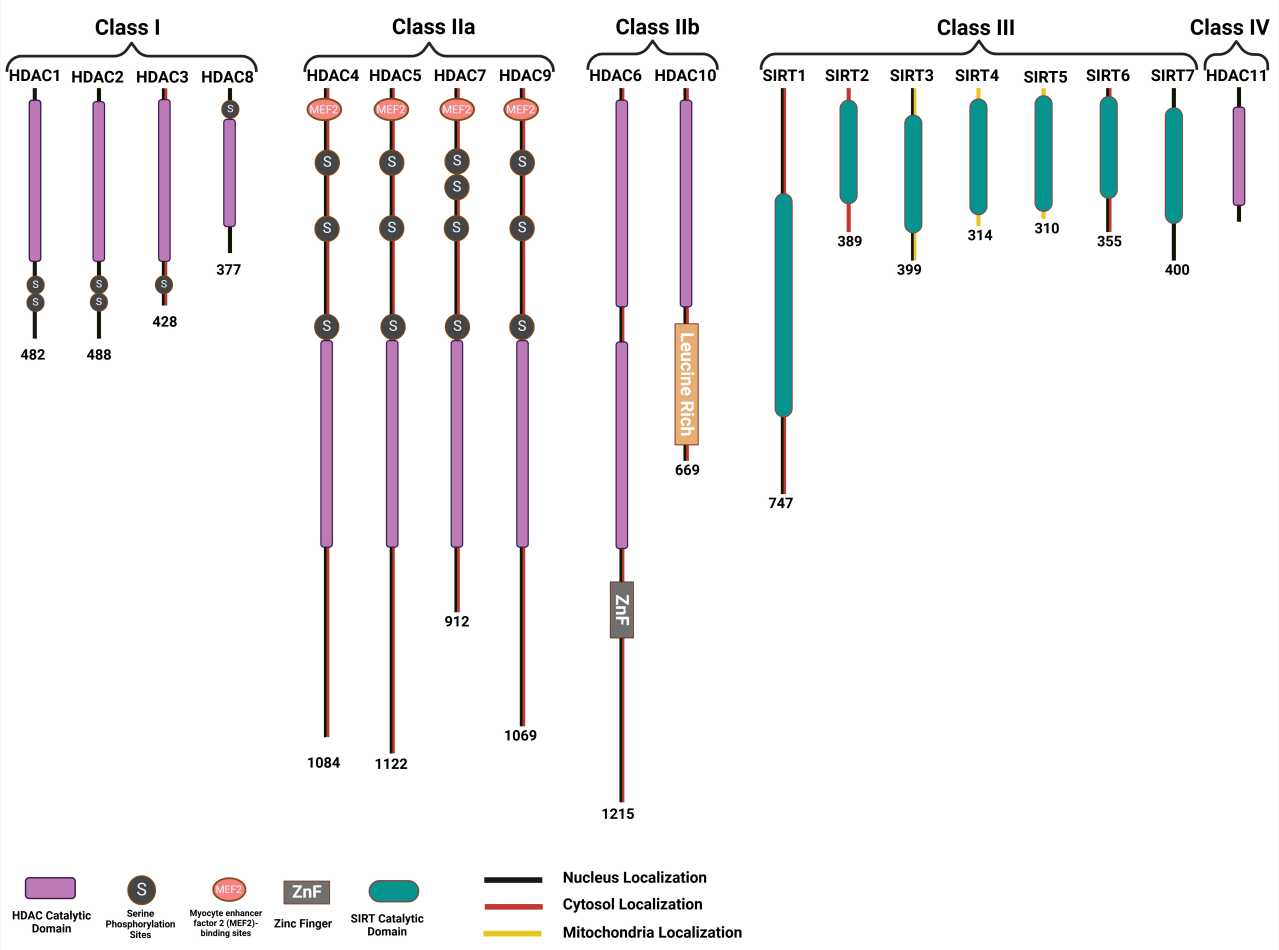
**Classifications of the histone deacetylase family and domains that characterize the different members of each HDAC subfamily**. HDAC, Histone deacetylase; SIRT, Sirtuin.

Histone deacetylases and sirtuins are enzyme families that modify histones by removing acetyl groups, thereby influencing chromatin structure and gene expression. These modifications can take place at distinct amino acid residues on both canonical histone proteins and variant histones, such as H3.1, H3.3, H2A.Z, and macroH2A. Prior research has thoroughly examined the post-translational modifications of histones and their contributions to cardiomyocyte differentiation and heart development, particularly in the context of tissue regeneration. The role of histone deacetylases extends to multiple cellular signaling pathways and disease processes, positioning them as key regulators in hypertension, vascular conditions, arrhythmias, HF, and angiogenesis. The HDAC family is classified into four categories: class I HDACs, which are HDAC1, HDAC2, HDAC3, and HDAC8; class II HDACs, which include HDAC4, HDAC5, HDAC6, HDAC7, HDAC9, and HDAC10; class III HDACs, which are SIRT1 to SIRT7; class IV HDACs, represented by HDAC11 (Fig. 4) [54].

### 2.4 Roles and Targets of Class I HDACs in Heart Diseases

HDAC1 features a typical HDAC domain characterized by a central parallel β-fold, which is enveloped by various α-helices that are connected to the ring. This structure enables the enzyme to catalyze the deacetylation of both histone and non-histone substrates [55]. Furthermore, HDAC1 is essential for modulating gene expression in a wide array of cellular functions, including cell senescence, autophagy, inflammation, and apoptosis, through the utilization of post-transcriptional and post-translational modification mechanisms.

The involvement of HDAC2 in cardiac disease was initially identified as a contributing factor to hypertrophy derived from the “homeodomain-only protein” (HOPX) [56]. HDAC1 and HDAC2 redundantly manage the regulation of cardiac development in the embryo. Cardiac-specific knockout of either HDAC1 or HDAC2 does not alter the process of cardiac morphogenesis. However, the double knockout of both HDAC1 and HDAC2 results in lethality by day 14 postnatally, caused by severe dilated cardiomyopathy. Furthermore, HDAC2 is recognized as the major class I HDAC in the hearts of adult organisms [56].

HDAC3 typically acts as a corepressor through deacetylating histone tails. The deletion of HDAC3 in cardiac tissue results in diminished cardiac contractility and increased lipid accumulation; however, the specific molecular role of HDAC3 in cardiomyopathy remains unclear. In the development of cardiac structures, the absence of HDAC3 in cardiac progenitor cells leads to disruptions in the development of the secondary heart field [57], the morphogenesis of lymphovenous valves, and the specification of cardiomyocyte lineages [58]. The involvement of EZH2 and HDAC3 in regulating the endothelial-to-mesenchymal transition (EMT) highlights their potential as effective epigenetic targets for counteracting TGF-β-induced EMT [59, 60]. *KAT2B* functions as an essential histone acetyltransferase epigenetic factor in the TGF-β signaling pathway, and variations in this gene are correlated with the etiology of CVDs [61]. Mouse embryos that do not express the chromatin-modifying enzyme HDAC3 in cardiac progenitor cells exhibit premature differentiation of cardiomyocytes, severe cardiac development defects, an increase in *TBX5* target gene expression, and embryonic lethality. HDAC3 forms a physical interaction with *TBX5*, modulating its acetylation to inhibit the *TBX5*-dependent activation of genes associated with the cardiomyocyte lineage [62].

HDAC8 is implicated in pathways that promote inflammation and fibrosis, and its activity is reliably upregulated in a cardiac model experiencing pressure overload due to thoracic aortic contraction (TAC). The overexpression of HDAC8 mechanically facilitates cardiomyocyte hypertrophy, whereas the selective inhibition of HDAC8 through PCI-34051 diminishes the activation of p38 mitogen-activated protein kinase (MAPK) following isoproterenol induction. Consequently, PCI-34051 has the potential to mitigate myocardial hypertrophy and fibrosis by modulating the p38 MAPK pathway [63].

### 2.5 Roles and Targets of Classes II and IV in Heart Diseases

HDAC4 functions as a zinc-dependent enzyme that facilitates the condensation of nucleosomes. Furthermore, HDAC4 interacts with transcription factors, thereby influencing cell proliferation, senescence, and differentiation. The zinc-dependent histone deacetylase HDAC4 plays a crucial role in regulating both the proliferation and apoptosis of human vascular endothelial cells associated with atherosclerosis [64]. The contrasting actions of CaMKII and PKA regulate the nuclear localization of HDAC4 in cardiomyocytes, whereby CaMKII promotes the export of HDAC4 from the nucleus. In contrast, PKA encourages the retention of HDAC4 through phosphorylation at S265/266. In the event of HF, the effect of CaMKII becomes predominant, leading to modifications in the transcriptional control exerted by HDAC4 [65].

HDAC5 has been identified as a regulator of the myocyte enhancer factor 2 (*MEF2*) family, which plays a vital role as a transcription factor in the cardiac growth and remodeling processes. It has been proposed that the nuclear export of HDAC5 responds to hypertrophic stimuli, contributing to pathological hypertrophy; therefore, inhibiting HDAC5 could be a viable approach to counteract cardiac hypertrophy (CH) [66].

Research indicates that HDAC7 is essential for maintaining vascular integrity during heart development; meanwhile, the role of HDAC4 in triggering vascular calcification has also been recognized. In addition, the possibility that SIK1 promotes CH by targeting HDAC7 was considered since a notable decrease in HDAC7 levels was observed when SIK1 was either absent or pharmacologically inhibited [67].

The class II histone deacetylase family includes HDAC9, which is commonly overexpressed in the tissues of the brain and heart muscles. The suppression of HDAC9 leads to a reduction in calcification and an increase in cell contractility, both of which serve as significant independent predictors of future cardiovascular events [68].

HDAC6, a class IIb cytoplasmic deacetylase comprising 1215 amino acids, has been widely reported in the literature since 1990. HDAC6 is capable of enhancing the emergence and evolution of CVDs along with corresponding tumors [69]. As a member of the class IIb category of the HDAC protein family, HDAC6 is distinguished by its role in histone deacetylation. Studies have shown that inhibiting HDAC activity can mitigate CH and fibrosis in various animal models that display hypertrophic conditions [70, 71]. HDAC6 is mainly localized in the cytoplasm and functions by deacetylating α-tubulin through its direct interaction with microtubules. Therefore, regulating HDAC6 is crucial for the meticulous regulation of α-tubulin acetylation in mammalian cells [69].

HDAC10, classified within the arginase/deacetylase superfamily, is expressed in numerous tissues, including the kidney, brain, pancreas, liver, heart, testis, spleen, and placenta [50]. Moreover, HDAC10 exists in both the nuclear and cytoplasmic compartments. Further, while HDAC6-deficient mice show a decrease in CH, the role of HDAC10 in the progression of CH remains unclear.

The structure of HDAC11 comprises a single lysine deacetylase domain, flanked by short N-terminal and C-terminal segments. Studies have shown that HDAC11 is primarily expressed in the heart, kidney, skeletal muscle, brain, and testes [72].

### 2.6 Roles and Targets of Class III in Heart Diseases

SIRT1 is the most recognized member of the sirtuin protein family, exhibiting widespread expression in numerous tissues, particularly in the vascular endothelium [73]. The inhibition of SIRT1 has been associated with vascular dysfunction and arterial thrombosis, along with modifications in fibrinolysis [73]. The role of miR-138-5p in HF involves promoting cardiomyocyte apoptosis by targeting and reducing SIRT1, thereby activating the p53 signaling pathway. Conversely, diminishing miR-138-5p levels can protect cardiac cells [74].

SIRT2 negatively influences heart health and contributes to the cardiac response to injury and the development of CH, thereby establishing this protein as a distinct member within the SIRT family. Meanwhile, deletion of SIRT2 is associated with reduced AMPK activation and an increase in CH related to aging and angiotensin II (Ang II) [75]. Moreover, advanced glycation end products and their receptors exacerbate diabetic cardiomyopathy by suppressing SIRT2 [76].

The human SIRT3 protein, consisting of 399 amino acids, is characterized by two main functional domains: a prominent Rossmann fold that features an NAD+ binding motif and a compact helical complex with a zinc-binding motif [77]. SIRT3 has been identified as a key factor in the heart, inhibiting the progression of CH, ischemia-reperfusion injury, cardiac fibrosis, and impaired angiogenesis, and safeguarding cardiomyocytes from cell death induced by oxidative stress [77]. SIRT3 is instrumental in mediating the often-complex profibrotic and proinflammatory activities of cardiac cells through its modulation of the FOS/AP-1 pathway [78].

The enzyme SIRT4, which has a molecular weight of 59 kDa and functions as an ADP-ribosyltransferase, is variably present in liver mitochondria and skeletal muscle and is associated with the regulation of homeostasis in glucose and lipid metabolism. The lack of SIRT4 plays a crucial role in significantly diminishing myocardial hypertrophy and fibrosis associated with Ang II infusion [79].

SIRT5 has been recognized as an essential factor in sustaining cardiac health and neuronal integrity during stress. The process of desuccinylation, mediated by SIRT5, is critical for maintaining energy metabolism in cardiac tissues. The function of SIRT5 in the cardiac stress response was examined through a well-established model of pressure overload-induced hypertrophy, specifically utilizing TAC. The absence of SIRT5 resulted in significant cardiac dysfunction following TAC, which correlated with an elevated mortality rate [80].

SIRT6 contributes to the maintenance of cardiac function through various roles, notably in protecting against oxidative damage, ischemia/reperfusion injury, and stimuli that induce hypertrophy. SIRT6 contributes positively to HF and the regulation of cardiac fibrosis, which is a significant pathological factor in the development of HF. SIRT6 serves to negatively regulate the differentiation of cardiac fibroblasts into myofibroblasts. Subsequently, the loss of SIRT6 results in increased proliferation of cardiac fibroblasts and enhanced extracellular matrix deposition, as well as the upregulation of genes linked to focal adhesion and fibrosis, mediated by NF-κB signaling [81]. SIRT7 stands out as the only sirtuin that is predominantly localized in the nucleoli, where it is responsible for the regulation of RNA polymerase I transcription. Furthermore, SIRT7 exhibits ubiquitous expression throughout the entire body [82].

## 3. The Role of Epigenetic Factors in Cardiac Physiology: Writers, Erasers, and Readers

### 3.1 Writers

**Fig. 5.**
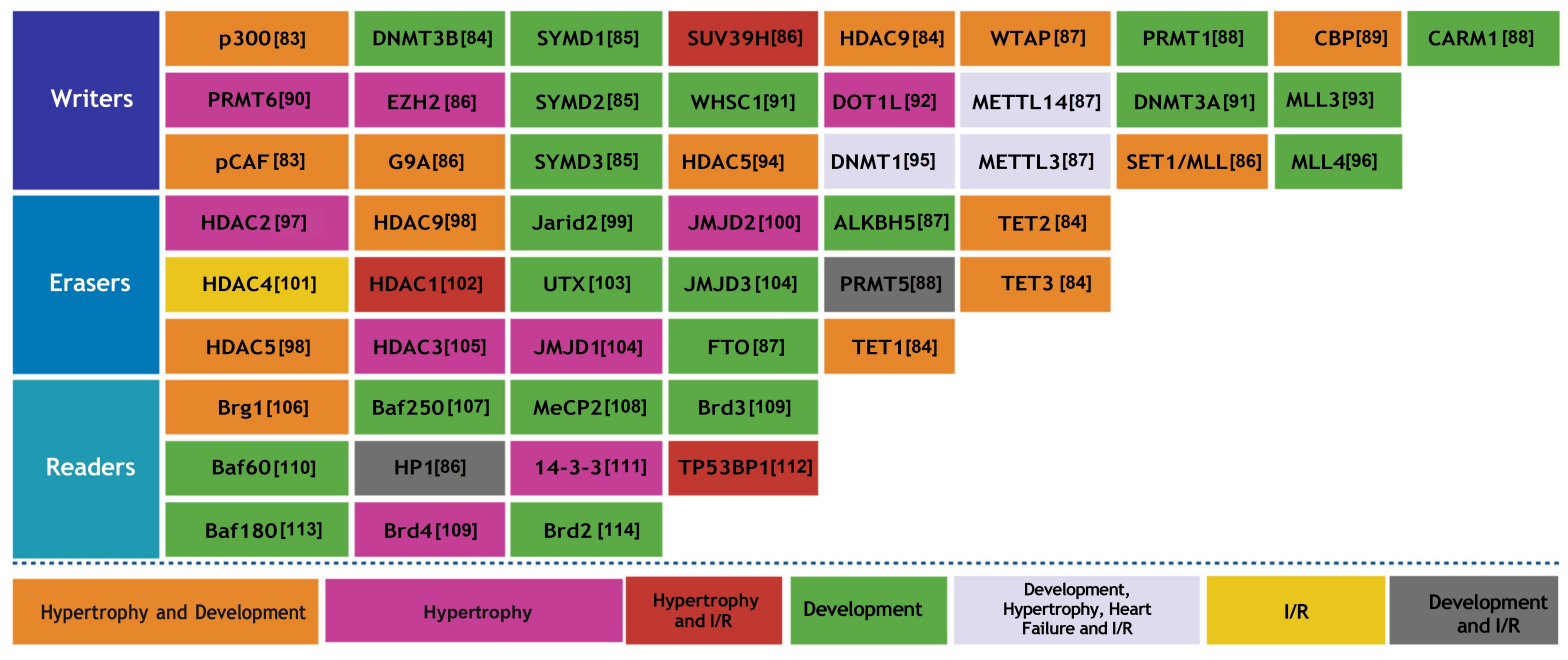
**The role of epigenetic factors, such as writers, erasers, and readers, is pivotal in the realm of cardiac physiology**. I/R, ischemia/reperfusion.

A variety of epigenetic writers, including histone acetyltransferases, methyltransferases, and RNA methylation regulators, are integral to cardiac development, homeostasis, and disease. When these enzymes are dysregulated, they can contribute to congenital heart defects, hypertrophy, ischemia/reperfusion injury, and HF by altering gene expression through chromatin and RNA modifications (Fig. 5) [83, 84, 85, 86, 87, 88, 89, 90, 91, 92, 93, 94, 95, 96, 97, 98, 99, 100, 101, 102, 103, 104, 105, 106, 107, 108, 109, 110, 111, 112, 113, 114].

Writers facilitate the attachment of chemical groups to DNA and histones, thereby influencing the regulation of gene expression. The function of erasers involves the removal of particular chemical groups, which in turn governs the activation or inhibition of genes. Readers can discern these modifications, allowing them to ascertain which genes are being expressed. These processes are fundamental to the development, operation, and diseases of the heart.

The role of the epigenetic regulator p300 is significant in both the development of the embryonic heart and the manifestation of heart disease in adults, as it influences gene expression through histone acetylation. Disruption of the function employed by p300 is associated with stress-induced cardiac aging and various related pathologies [115]. The expression of *GATA4* in embryonic mouse hearts was influenced by p300 through its regulation of histone acetylation. When p300 was downregulated via RNA interference, there was a notable change in the global acetylation of H3, along with the acetylation of H3K4, H3K9, and H3K27 at the promoters of *GATA4* and *TBX5* [116]. Cardiac-specific deletion of the DNMT3B in mice resulted in the rapid progression of HF, driven by myocardial thinning, fibrosis, and abnormal sarcomere configurations, without the occurrence of prior hypertrophy [117]. *SMYD1*, a muscle-specific histone methyltransferase, regulates early heart development by activating ISL1 through H3K4 trimethylation and repressing ANF expression via interaction with HDAC. Thus, the loss of *SMYD1* disrupts these regulatory mechanisms, leading to severe cardiac structural defects and embryonic lethality [118]. *SMYD2*, a histone methyltransferase, is highly expressed in the neonatal heart but is not essential for normal cardiac development or function in mice [119]. Myoblast function is compromised by chronic hypoxia due to the upregulation of HDAC9, which directly inhibits autophagy by repressing key genes associated with this process. This epigenetic disruption leads to the inactivation of the Wnt signaling pathway, resulting in the sustained dysfunction of muscle cells [120]. WTAP contributes to the proliferation and migration of cardiac fibroblasts in diabetic cardiac fibrosis by promoting mitochondrial lipid oxidation. This enhancement occurs through the m6A methylation-dependent degradation of AR, mediated by YTHDF2 [121]. Ischemia/reperfusion (I/R) injury negatively impacts SIRT1 transcription in the heart through the action of *SUV39H1*, which mediates the trimethylation of H3K9 at the SIRT1 promoter. Conversely, blocking *SUV39H1* can reverse this effect, leading to increased SIRT1 expression, smaller infarct size, and improved cardiac function [122]. This study elucidates a novel function of *CARM1* in heart development, as mutations in *CARM1* are linked to severe congenital heart defects in mice, notably including ventricular septal defects (VSDs) and persistent truncus arteriosus [123].

*PRMT6* is significantly upregulated in failing human hearts and promotes CH by increasing repressive H3R2Me2a marks and reducing H3K4Me3 [90]. EZH1 and EZH2 regulate skeletal growth by catalyzing H3K27 trimethylation, which controls chondrocyte proliferation and hypertrophy in the growth plate [124]. Insufficient levels of *WHSC1* cause growth delays and congenital malformations, including heart-related anomalies, underscoring its vital role in maintaining effective transcriptional regulation [125]. The practice of endurance exercise training diminishes cardiac *m6A* mRNA levels, while the downregulation of *METTL14* is linked to the physiological CH that occurs in response to exercise. *METTL14* is critical in regulating cardiac homeostasis [126]. Adult cardiac progenitor cells exhibit a constrained ability to regenerate, primarily due to the inhibition of the *WNT* antagonist *WIF1* through DNA methylation, a process governed *de novo* by the methyltransferase DNMT3A [127]. *MLL3*, a member of the mixed lineage leukemia (MLL) family, is significantly overexpressed in the hearts of individuals with dilated cardiomyopathy (DCM) and is correlated with detrimental markers of cardiac remodeling, including left ventricular diameter and ejection fraction [93].

pCAF is responsible for the acetylation of the fetal gene *ACTA1* in response to β-adrenergic activation, a process that contributes to left ventricular hypertrophy (LVH) and impairs cardiac function [128]. In cardiomyocytes, the sequential activation of BRG1, G9a/GLP, and DNMT3 results in the assembly of a complex that represses the *Myh6* gene, which is vital for cardiac contraction, through H3K9 and CpG methylation [129]. Zebrafish embryos exhibit *SMYD3* expression during all phases of their development. Inhibiting SMYD3 expression through antisense morpholino oligonucleotides caused marked developmental issues, including pericardial edema (fluid accumulation surrounding the heart) and an atypical trunk structure [130]. Mice deficient in HDAC5 develop hypertrophied hearts in response to pressure overload induced by aortic constriction or calcineurin activation, a phenotype similar to that observed in *HDAC9* knockout mice [131]. A regulatory interplay exists between DNMT1 and METTL3 in CH. DNMT1 reduces the expression of *METTL3*, which helps shield heart cells from stress-related apoptosis, whereas METTL3 is known to induce apoptosis in pathological environments [95]. *MLL4* plays a critical role in regulating endoplasmic reticulum (ER) stress and protecting against pressure overload-induced CH and HF [132].

### 3.2 Erasers

Epigenetic erasers are enzymes that eliminate chemical modifications from histones or DNA, effectively reversing epigenetic marks and influencing gene expression. In the heart, key enzymes including HDACs, JMJD3 demethylases, and TETs, play significant roles in regulating cardiac development, hypertrophy, and repair.

A decrease in HDAC2 levels in mice resulted in a lower sensitivity to hypertrophic stimuli; in contrast, mice with elevated HDAC2 expression experienced CH [133]. MEG3 modulates CH by acting as a ceRNA that regulates the expression of miR-361-5p and HDAC9, with its upregulation induced by the transcription factor STAT3 [134]. Hyperglycemia leads to a reduction in nitric oxide (NO) production, which subsequently raises *JARID2* expression and inhibits *NOTCH1* below the critical threshold needed for healthy heart development [135]. *JMJD2A*, known as a histone demethylase, promotes the occurrence of pathological CH by stimulating *FHL1* expression through the demethylation of H3K9 and its association with SRF and myocardin. The expression of *JMJD2A* is notably increased in both stressed mouse hearts and human hypertrophic cardiomyopathy [100]. *ALKBH5*, a demethylase of m6A, plays a vital role in the regeneration of heart tissue. Enhancing the expression of *ALKBH5* supports the proliferation of cardiomyocytes and leads to improved cardiac performance following injury [136]. TET2 and TET3 are critical for the early stages of cardiac development, functioning to regulate DNA demethylation and chromatin structure. The absence of these enzymes leads to ventricular non-compaction cardiomyopathy and adversely affects cardiomyocyte differentiation and gene expression [137].

The activation of HDAC4 has been identified as a significant factor in myocardial ischemia/reperfusion injury. Overexpressing active HDAC4 specifically in cardiomyocytes exacerbated cardiac damage and hindered recovery, whereas inhibiting HDAC alleviated these adverse effects [138]. UTX, an enzyme that demethylates H3K27, is essential for activating cardiac genes during the development of the heart. Thus, a deficiency in UTX results in impaired cardiac differentiation and can cause embryonic lethality. UTX enhances heart-specific gene expression by demethylating H3K27 and aiding in the recruitment of BRG1 to cardiac enhancers [103]. *ISL1* is instrumental in the demethylation of H3K27me3 at the enhancers of key cardiac genes by recruiting the demethylase *JMJD3*. The lack of either *ISL1* or *JMJD3* compromises differentiation, thereby illustrating their important collaboration in cardiac lineage commitment [139].

### 3.3 Readers

Epigenetic readers are crucial in recognizing and binding to certain histone modifications to control gene expression. In the heart, proteins such as BRD4 and BRG1 act as readers that modulate stress responses and cardiac remodeling by analyzing chromatin signals.

BRG1, a fundamental enzyme in the SWI/SNF chromatin remodeling complex, is a major epigenetic regulator associated with CVDs, overseeing the regulation of gene expression and protein levels [140]. BAF250a is responsible for directing the expression of vital cardiac genes, such as *MEF2C*, *NKX2.5*, and *BMP10*, by transporting Brg1 to their promoters, which increases chromatin accessibility and transcriptional activation and ensures proper heart development [141]. MeCP2 appears to play a critical role in HF by modulating genes (*MYH6*, *JAK1*, *DECR1*, *SETD1B*, *LYZ2*, *ALOX5AP*, *TTN*, *TPM3*, *HRC*, and *MYH11*) involved in cardiac structure and immune responses [142].

Baf60c regulates the expression of genes critical for sarcomere integrity, cardiac metabolic processes, and contractile capabilities, with a significant number of these genes being regulated by *MYOCD*, a cofactor of both *MEF2* and *SRF* [143]. The role of HP1γ in cardiac myocytes has been clarified, demonstrating that, although HP1γ is not required for cell cycle exit or cardiac growth, it significantly contributes to epigenetic regulation by ensuring the maintenance of H4K20me3, an essential heterochromatin marker [144]. The interaction between 14-3-3 proteins and HDAC4 and HDAC5, both of which are histone deacetylases involved in muscle differentiation and remodeling, is noteworthy. In the presence of signaling pathways, such as calcium/calmodulin-dependent kinase (CaMK), 14-3-3 proteins bind to HDAC5, resulting in its nuclear export and promoting the expression of muscle genes that support hypertrophic growth [145].

BAF180 ablation hinders the EMT and causes atypical formation of coronary vessels, which is associated with the downregulation of vital signaling pathways, including *FGF*, *TGF*, and vascular endothelial growth factor (VEGF) [146]. BRD4 functions by modulating abnormal cardiac gene expression under stress conditions. BRD4, recognized as a chromatin ‘reader’, is generally inhibited by miR-9; however, stress signals result in the downregulation of miR-9, which subsequently enhances BRD4 binding to super-enhancers linked to pathological cardiac genes [147]. Therefore, BRD2 levels increase when β-adrenergic agonists induce hypertrophy, and the overexpression of BRD2 encourages hypertrophic changes, while silencing it prevents hypertrophy. The regulation of metabolic genes by BRD2, particularly those linked to the TCA cycle, contributes to the enhancement of cardiac metabolism [148].

## 4. Heart Diseases

### 4.1 CVD and Arrhythmia

Familial heart disease is responsible for significant morbidity and mortality worldwide. The regulation of cardiac disease-related gene function and expression is predominantly influenced by epigenetic factors, which involve DNA methylation, histone modification, and ncRNA regulation, ultimately affecting the progression of heart diseases. There are three categories of proteins that play crucial roles in the context of DNA and histone methylation marks, specifically writers, erasers, and readers. Risk factors such as smoking, age, obesity, hypertension, and diabetes predispose individuals to the development of CVD. Many monogenic inherited forms of cardiomyopathy, aortic aneurysm, and ion channelopathy have been clinically reported. Meanwhile, the molecular basis of epigenetics, as a form of inheritance, has been studied in a variety of organisms. A genetic factor has been recognized in many cases of CVD that were previously thought to be idiopathic, with multiple genes now evidently connected to monogenic variants of CVD [149]. A study identified five genes (*ATG7*, *BACH2*, *CDKN1B*, *DHCR24*, and *MPO*) that are regulated by DNA methylation and are essential in the context of coronary heart disease, utilizing machine learning models that incorporate both methylation and expression data [150].

Most clinical investigations conducted up to this point have centered on genes implicated in hereditary CVDs among patients and their families suffering from arrhythmias and sudden cardiac mortality [151]. The knockout of TETs leads to the hypermethylation of gene promoters that encode WNT inhibitors, resulting in hyperactivation of WNT signaling and subsequent defects in cardiac mesoderm patterning [152]. This focus has led to a detection bias favoring clinically affected individuals and their relatives, which may impact the accuracy of gene penetrance estimates for genes associated with the disease. The involvement of lactylation in heart development following birth is underscored by the rise in non-histone lactylation levels observed from 1 to 6 weeks postpartum. In particular, the lactylation of histone 4 at lysine 12 (H4K12la) is a critical factor in regulating gene expression during the early stages of cardiac development [153]. Recently, studies from population-based cohorts have been published that lack certainty over clinical symptoms or criteria [154]. Alcohol led to a reduction in histone H3K9me3 methylation by influencing the expression of G9α, a histone methyltransferase in cardiomyocytes. Additionally, an overexpression of cardiomyogenesis-related genes, including *MEF2C*, *CX43*, *ANP*, and β*-MHC*, was detected in the hearts of fetal mice that were exposed to alcohol [155]. The initial analysis of Long Interspersed Nuclear Element-1 (LINE-1) indicated that patients with CVD exhibited reduced levels of methylation. *NOS3* is an endothelial gene pertinent to angiogenesis and CVD, regulated through histone modifications. *NOS3* encodes endothelial nitric oxide synthase (eNOS), which catalyzes the synthesis of nitric oxide from L-arginine within the vascular system [156, 157].

### 4.2 Coronary Artery Disease (CAD)

Research utilizing LINE-1 has demonstrated a notable correlation between reduced methylation levels and elevated systolic and diastolic blood pressures. Hypomethylation at ALU elements has been linked to increased blood pressure [158]. A prior study identified 34 CpG sites associated with acute myocardial infarction (AMI) through an epigenome-wide association study, highlighting the roles of smoking, lipid metabolism, and inflammation. Four of these sites were further connected to coronary heart disease and CVD, although these findings did not improve the predictive performance of existing risk models [159]. Hypomethylation at LINE-1 is inversely related to the incidence of CAD and stroke. Conversely, there appears to be an association between global hypermethylation of LINE-1 and the vascular inflammatory response to damage sustained by the endothelium. It has been established that hypomethylation of LINE-1 correlates with an increased likelihood of Tetralogy of Fallot (TOF) in infants. Methylation of cytosine within the *IGF2* gene leads to the dysregulation of imprinting, which is linked to an increased risk of coronary heart disease [160]. Significant changes in the expression of 61 miRNAs and 135 small nucleolar RNAs (snoRNAs) were noted in the myocardium of children with TOF compared to those in normally developing subjects. Moreover, researchers identified 229 genes crucial for heart development, with 44 of these genes showing significant changes in expression in the myocardium of individuals with TOF compared to those in a typically developing myocardium [161]. The expression of miR-421 was likewise noted to significantly negatively correlate with SOX4, a principal regulator of the NOTCH pathway, which has been established as crucial for the development of the cardiac outflow tract [162].

In a comparative analysis, 4720 genes were found to be differentially methylated between patients with chronic Chagas cardiomyopathy and control subjects, with 399 of these genes also demonstrating differential expression. Among these, several genes are linked to cardiac function and contain methylation sites in their promoter regions. These include the potassium channel genes *KCNA4* and *KCNIP4*, which are implicated in electrical conduction and arrhythmias, as well as *SMOC2*, which is associated with matrix remodeling. Enkephalin and *RUNX3* may play a role in the exacerbated inflammatory damage in the heart, mediated by T-helper 1 cytokines [163].

### 4.3 Cardiomyopathy

Prior research has highlighted four key genes (*CREBBP*, *PPP2R2B*, *BMP4*, and *BMP7*) as potential regulators of DCM associated with *LMNA* mutations, which function through the WNT/β-catenin and TGFβ-BMP pathways. These findings reveal that disruption of interactions between *LMNA* or *LAP2α*–Lamin A/C complexes and euchromatin may be a contributing factor to the DCM pathogenesis [164]. The analysis showed that the differential DNA methylation observed between non-failing hearts and those in end-stage failure was not uniformly distributed throughout the genome; rather, the differential DNA methylation was predominantly localized to promoter CpG islands, intragenic CpG islands, and gene bodies. In a recent study that applied capture-based bisulfite sequencing methods, researchers identified 151 regions exhibiting differential methylation in DCM relative to non-failing hearts [165]. A loss of miR-22 rendered mice more prone to the onset of dilated cardiomyopathy under stressful conditions, with SIRT1 and HDAC4 identified as targets of miR-22 in the cardiac environment. Additionally, miR-22 was found to be essential for supporting hypertrophic cardiac growth in response to stress [166]. Significant changes in the mRNA expression of *LY75* and *ADORA2A* were found to be associated with aberrant DNA methylation in individuals suffering from DCM. The corresponding genes *LY75* and *ADORA2A* in zebrafish suggest that these genes play a crucial role in the adaptive or maladaptive pathways involved in HF. The study found 59 CpG loci that exhibited significant variations in DNA methylation in the myocardium of individuals with DCM compared to clinical controls. Of these loci, 29 were hypomethylated, while 30 were hypermethylated in the DCM group [167]. The *CTNIR193H* mutation contributes to the development of restrictive cardiomyopathy by interacting with HDAC1, which leads to the deacetylation of histones at the *PDE4D* promoter and the suppression of its expression [168].

Interestingly, 532 of the 664 examined miRNAs were found to be expressed in at least one heart sample. Among these, 13 miRNAs exhibited differential expression in hypertrophic cardiomyopathy when compared to donor samples. The genomic analysis of these differentially expressed miRNAs indicated that miR-204 is situated within an intron of the *TRPM3* gene, which encodes a non-specific cation channel that plays a role in calcium influx [169].

### 4.4 Valvular Heart Disease (VHD)

A deeper comprehension of the role of epigenetic regulations in VHD may provide a fresh perspective for translational research aimed at developing new diagnostic tools and innovative approaches in drug design and discovery. A correlation has been established between the hypermethylation related to hydroxymethylation of the *EGFR* gene and the occurrence of aortic valve calcification, which subsequently leads to ventricular hypertrophy [170]. ALKBH5, an m6A demethylase, plays a significant protective role in the context of calcific aortic valve disease by diminishing m6A modifications on *TGFBR2* mRNA. This reduction leads to the inhibition of the TGFBR2/SMAD2 signaling pathway and the calcification of valve interstitial cells [171]. According to Hiltunen and associates [172], the presence of global DNA hypomethylation was first documented in advanced atherosclerotic lesions found in humans, mice, and rabbits. A comprehensive analysis of DNA methylation across the genome, conducted on neonatal dried blood spots, revealed significant alterations in CpG methylation at 59 locations within 52 genes associated with aortic valve stenosis [173]. In a study of methylation patterns in the placentas of eight term subjects with isolated VSD compared to ten unaffected controls, researchers found 80 CpG sites and eight microRNAs that showed promise in accurately detecting VSD. The analysis revealed 36 miRNAs that were differentially expressed in patients with VSD compared to those without VSD. The predominant target genes were mainly associated with the morphogenesis of the cardiac right ventricle. Additionally, *NOTCH1*, *HAND1*, *ZFPM2*, and *GATA3* were identified as potential targets of hsa-let-7e-5p, hsa-miR-222-3p, and hsa-miR-433 [174]. The loss of SERCA2 results in dysfunction during both the systolic and diastolic phases, and the buildup of sodium ions further disrupts relaxation [175]. *PTEN* is identified as a direct target of miR-132/212 and may be implicated in the cardiac response to these microRNAs. Moreover, miR-132/212 was found to be upregulated in cases of end-stage HF, which correlates with a reduction in SERCA2a expression [176]. The upregulation of calcium extrusion mechanisms can help maintain diastolic function near normal in the absence of *SERCA2*.

### 4.5 Ischemic Injury, Myocardial Infarction (MI), and Atherosclerosis

Individuals with diminished LINE-1 methylation were found to have a heightened risk of ischemic heart disease and stroke. The failing mammalian heart and hypoxic cardiomyocytes exhibit a decrease in *FTO* expression, which correlates with an increase in m6A in RNA and a reduction in the contractile function of cardiomyocytes. The ischemia-induced elevation in m6A and the subsequent decline in cardiac contractile function were alleviated by augmenting *FTO* expression in failing mouse hearts [177]. Additionally, miR-132 in cardiomyocytes effectively reduced the increase in intracellular Ca^2+^ concentrations under hypoxic stress. Moreover, treatment with exogenous miR-132 diminished the expression of apoptotic factors, including Bax, cytochrome C, and caspase 3, reducing the number of apoptotic cells. This microRNA specifically targets *NCX1*, whose expression is heightened during hypoxia [178]. In a subsequent study, researchers deleted a regulatory DNA element from the mouse genome and investigated its role in regulating *Cacna1g* expression within the cardiac conduction system. This deletion also revealed a *TBX3*-dependent gene regulatory network in the atrioventricular conduction system, which contributes to the electrophysiology of the heart [179]. The regulation of cardiac injury and dysfunction resulting from I/R is significantly influenced by miR-320, which exerts opposing effects on *HSP20* [180]. Hypermutability in various cardiac genes, such as the cardiac isoform of *MYBPC3*, can be attributed to DNA methylation, which shows a higher extent of exonic methylation at CpG sites compared to the skeletal muscle isoform [181]. Indeed, miR-122 facilitates EMT both *in vitro* and *in vivo*, while the suppression of miR-122 partially inhibits the EMT process triggered by H_2_O_2_ by regulating *NPAS3* expression [182]. YAP/TAZ facilitates a proinflammatory response by upregulating IL-6 expression and inhibits the reparative response by downregulating Arg1 expression. This mechanism involves their interaction with the HDAC3–NCoR1 repressor complex, resulting in decreased fibrosis and hypertrophy, increased angiogenesis, and ultimately enhanced cardiac function post-MI [183].

The investigation of DNA methylation in the early phase of AMI has yielded encouraging results, highlighting epigenetic biomarkers linked to the promoter methylation of five genes: *CSF1R*, *MAP3K14*, *PTPN6*, *COL6A1*, and *CYBA*. These biomarkers may prove valuable for early clinical diagnosis and as targets for AMI treatment [184]. An age-related increase in DNA hypermethylation within mitochondrial genes reduced the expression of key genes (*CYT B*, *ND1*, *ND6*, *ND4L*, *COX1*, *COX2*, and *ATP8*), which are essential for cardiac contractility, thereby increasing the susceptibility of adult hearts to I/R injury [185]. The development of myocardial fibrosis in Ang II-dependent hypertension is governed by the downregulation of miR-133a and miR-29b, impacting the expression levels of Col1A1 [186]. Fifteen years prior, pioneering genome-wide research focused on DNA methylation in the failing human myocardium was undertaken. This research first indicated that a substantial proportion of CpG islands and promoters were hypomethylated in the heart at the end stage of failure, with these distinct DNA methylation patterns correlating with variations in the expression of angiogenic factors. The enhancement of the m6A modification of pri-miR-19a by *METTL14* facilitates its maturation, promoting the proliferation and invasion of endothelial cells associated with atherosclerosis. Thus, this METTL14/m6A/miR-19a pathway may serve as a promising target for therapeutic development in atherosclerosis [187]. The upregulation of miR-138 is significant for the protective adaptation of myocardial tissue to chronic hypoxia, mainly mediated by the MLK3/JNK/c-Jun signaling pathway [188].

### 4.6 HF

Overexpression of miR-24 reduced TGF-β secretion and *SMAD2/3*phosphorylation in cardiac fibroblasts. Additionally, furin, a protease that regulates the activation of latent TGF-β, was identified as a potential target of miR-24 in the context of fibrosis [189]. Moreover, alterations in DNA methylation and corresponding changes in gene expression were identified in HF tissue with varying etiologies. A comprehensive study of 195 unique differentially methylated regions found one hypermethylated region (*HEY2, COX17, MSR1*, and *MYOM3*), two hypomethylated regions (*MMP2* and *CTGF*), and two miRs: one hypermethylated (miR-24-1) and one hypomethylated (miR-155) [165]. The optimized miR-132 inhibitor, referred to as anti-miR-132, has proven to be effective, safe, and well-tolerated in several models, including pigs, highlighting its potential as a new therapeutic option for HF [190]. Whole-genome methyl-binding domain-capture sequencing was employed to identify differentially methylated DNA regions in patients experiencing severe CAD alongside varying degrees of HF. Significant candidate biomarkers, including *HDAC9*, *JARID2*, *GREM1*, and *PDSS2*, were identified, indicating that DNA methylation may contribute to the classification of HF phenotypes [191]. The identification of epigenetic biomarkers within CD4+ T cells is essential for distinguishing HF with preserved ejection fraction (HFpEF) from various other forms of HF. Employing high-resolution DNA methylation analysis, particularly through reduced representation bisulfite sequencing, has led to the discovery of specific gene markers, namely *FOXB1*, *ELMOD1*, *DGKH*, *JUNB*, *SETD7*, and *MEF2D*, which improve the accuracy of diagnostics. The SETD7–RELA–IL6 pathway, in particular, has shown strong predictive capabilities in overweight or obese patients who are affected by HFpEF [192]. A total of 18 studies focusing on candidate genes have identified 16 genes with differential methylation [193]. This includes genes related to the renin–angiotensin system, such as the *ACE* promoter and *AGTR1*, as well as those that participate in maintaining sodium homeostasis and controlling extracellular fluid volume, including the *NET* promoter, *SCNN1A*, and *ADD1*. Meanwhile, *HTRA3*, a serine peptidase 3, is instrumental in maintaining the dormant state of cardiac fibroblasts by degrading TGF-β. When pressure overload leads to a decrease in HTRA3 levels, it activates the TGF-β signaling pathway, potentially resulting in fibrosis and HF [194]. Among the three epigenome-wide association studies (EWAS) conducted, hypertensive patients exhibited lower methylation levels of *SULF1*, *SKOR2*, and *EHMT2* when compared to normotensive individuals. The dysfunction of the *NET* gene has been previously associated with postural orthostatic tachycardia syndrome, which is characterized by a documented coding mutation in the *NET* gene [195]. In sepsis-induced myocardial dysfunction, the increased levels of *MALAT1* contribute to cardiomyocyte apoptosis by attracting EZH2, which subsequently inhibits the expression of USP22 [196]. Selenium supplementation acts as a protective agent against HF resulting from advanced glycation end-products by decreasing oxidative stress and preventing myocyte apoptosis. Furthermore, selenium restores *GPX1* expression by inhibiting the DNA methylation of the *GPX1* promoter that is mediated by DNMT2 [197]. Four specific microRNAs (miRs 34a, 28, 148a, and 93) showed increased expression levels in right ventricular hypertrophy/failure (RVH/RVF); meanwhile, these microRNAs were either downregulated or exhibited no change in left ventricular hypertrophy/failure (LVH/LVF) [198].

### 4.7 Congenital Heart Disease (CHD)

CHD refers to the functional and structural anomalies of the heart and vascular system that occur during embryonic development. Additionally, CHD stands as the primary contributor to mortality rates among perinatal and infant populations. The incidence of CHD at birth varies considerably across various countries and continents, with a commonly accepted prevalence rate of around 8 per 1000 live births [199]. The regulation of gene expression during heart development is effectively managed by numerous critical transcription factors, including *GATA4*, *TBX5*, *HAND2*, *MEF2C*, *NKX2.5*, and microRNAs (miR-1, miR-133, miR-208, and miR-499). In patients diagnosed with TOF, the methylation levels of *NKX2.5* and *HAND1* were markedly higher than those observed in the control subjects [200]. The transcription factor *NKX2.5* plays a crucial role in regulating second heart field progenitors, which are essential for the formation of the outflow tract and are associated with CHD in humans. A group of genes, namely *LRRN1*, *ELOVL2*, *SAFB*, *SLC39A6*, *KHDRBS1*, *HOXB4*, *FEZ1*, *CCDC117*, *JARID2*, *NRCAM*, and *ENPP3*, are expressed in second heart field (SHF) and pharyngeal arch tissues, with their regulation being contingent upon *NKX2.5* [201]. The impairment of S-nitrosylation-mediated regulation of GRK2 in aging GRK2-C340S mice promotes cardiovascular issues, including diminished cardiac function, fibrosis, and maladaptive hypertrophy [202]. A previous investigation indicated a substantial increase in the methylated promoter region of the *MTHFR* gene in mothers of individuals with Down syndrome and CHD, relative to other cohorts. This observation emphasizes the relationship between *MTHFR* promoter hypermethylation and mothers of children with Down syndrome exhibiting cardiac defects [203]. Differential methylation was observed in multiple genes associated with heart development and postnatal heart disease in the blood DNA of newborns with TOF. These genes include *RUNX*, *ABCB1*, *SELL*, *PPP2R5C*, *CREM*, *TLR1*, *SCN3A*, and *LHX9* [204]. The protein EZH2 functions as the key histone methyltransferase in the polycomb repressor complex 2. *EZH2* mutant mice exhibited a wide range of cardiovascular malformations that ultimately resulted in perinatal death. The endocardial cushions were underdeveloped in these mutants, and the EMT process was compromised [205]. Certain DNA methylation modifications in placental tissue can be indicative of TOF. The investigation of 165 differentially methylated CpG loci identified biomarkers with strong predictive capabilities, and pathway analysis emphasized the dysregulation of gene pathways that are integral to cardiac development, especially in relation to CH [206]. The study revealed notable alterations in methylation patterns of specific genes among children diagnosed with CHD and extracardiac malformations. Specifically, hypermethylation of *SNRPN* and *ZAC1*, along with hypomethylation of *INPP5F*, was associated with a heightened risk of disease [207].

### 4.8 CH

The induction of hypertrophy is mediated by calcineurin, a calcium-dependent serine/threonine protein phosphatase, which functions through the transcription factor *NFATC3* [208]. Twinfilin-1, a regulatory protein of the cytoskeleton, is a target of miR-1. When hypertrophic stimuli reduce miR-1 levels, this results in the upregulation of twinfilin-1, which subsequently promotes hypertrophy through its effects on the cardiac cytoskeleton [209]. The downregulation of *NRON* occurs in response to hypertrophic stimuli, and its increased expression intensifies hypertrophy. In a mouse model subjected to TAC to induce hypertrophy, the overexpression of *NRON* specifically in cardiomyocytes aggravated the condition, whereas the deletion of *NRON* mitigated it [210]. Ang II is responsible for the downregulation of miR-30 in cardiomyocytes, which consequently leads to myocardial hypertrophy via excessive autophagy [211]. MEOX1, a transcription factor specifically found in activated fibroblasts, interacts with likely regulatory elements of a wide-ranging fibrotic gene program and is necessary for the activation of fibroblasts triggered by TGF-β [212]. The long-term loss of normal cardiac structure and function *in vivo* is attributed to the reduction in m6A levels resulting from the deletion of *METTL3* in cardiomyocytes. Notably, the enhancement of *METTL3* levels resulted in a marked increase in cardiomyocyte size, demonstrating that *METTL3* expression alone can effectively induce cardiomyocyte hypertrophy [213]. JMJD1C, which functions as an H3K9me2-specific histone demethylase, is instrumental in the process of CH and fibrosis triggered by Ang II by promoting the transcription of *TIMP1* [214].

## 5. Non-Coding RNAs

Long non-coding RNA constitutes 68% of the total transcribed RNA. Short non-coding RNAs, commonly referred to as short ncRNAs, are RNA transcripts that typically consist of approximately 200 nucleotides. Notable examples of these include microRNAs, which range from 19 to 23 nucleotides; short interfering RNAs measuring between 21 and 25 base pairs; transfer RNAs that span 74 to 95 nucleotides; endogenous RNAs; small nuclear RNAs of about 100 base pairs; small nucleolar RNAs ranging from 100 to 300 base pairs; piwi-interacting RNAs, which are 24 to 30 base pairs in length and play a role in the negative regulation of gene expression [215].

Long non-coding RNAs consist of intergenic sequences, transcripts that may intersect with other coding regions in either the sense or antisense direction, in addition to enhancer RNAs. These enhancer RNAs function over considerable distances and across different chromosomes to facilitate the activation of transcription at remote promoters [215, 216].

### Roles of MicroRNAs in Heart Diseases

**Fig. 6.**
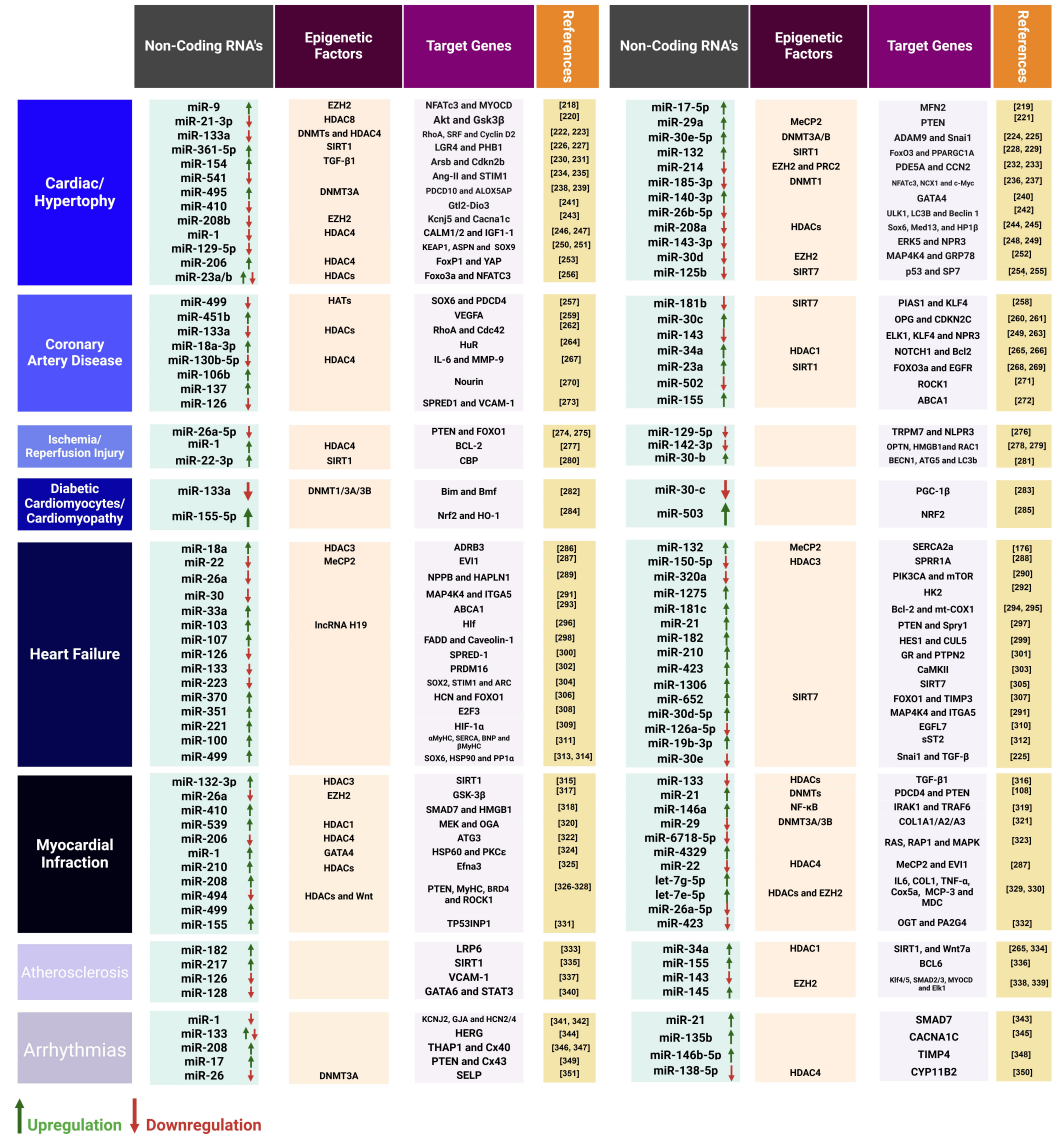
**The regulation of microRNAs (miRNAs) in cardiac tissue is instrumental in the prevention of heart diseases**.

The transcription of microRNA from miRNA loci and their corresponding host genes indicates the participation of transcription factors, epigenetic regulators, and enhancers, which are key components of the transcriptional machinery [217]. Variations in miRNA expression are typically presented (Fig. 6, Ref. [108, 176, 218, 219, 220, 221, 222, 223, 224, 225, 226, 227, 228, 229, 230, 231, 232, 233, 234, 235, 236, 237, 238, 239, 240, 241, 242, 243, 244, 245, 246, 247, 248, 249, 250, 251, 252, 253, 254, 255, 256, 257, 258, 259, 260, 261, 262, 263, 264, 265, 266, 267, 268, 269, 270, 271, 272, 273, 274, 275, 276, 277, 278, 279, 280, 281, 282, 283, 284, 285, 286, 287, 288, 289, 290, 291, 292, 293, 294, 295, 296, 297, 298, 299, 300, 301, 302, 303, 304, 305, 306, 307, 308, 309, 310, 311, 312, 313, 314, 315, 316, 317, 318, 319, 320, 321, 322, 323, 324, 325, 326, 327, 328, 329, 330, 331, 332, 333, 334, 335, 336, 337, 338, 339, 340, 341, 342, 343, 344, 345, 346, 347, 348, 349, 350, 351]). This figure provides a comprehensive overview of miRNA-mediated regulation in heart diseases, aiding in the identification of potential therapeutic targets and molecular pathways involved in heart-related disorders [108, 176, 218, 219, 220, 221, 222, 223, 224, 225, 226, 227, 228, 229, 230, 231, 232, 233, 234, 235, 236, 237, 238, 239, 240, 241, 242, 243, 244, 245, 246, 247, 248, 249, 250, 251, 252, 253, 254, 255, 256, 257, 258, 259, 260, 261, 262, 263, 264, 265, 266, 267, 268, 269, 270, 271, 272, 273, 274, 275, 276, 277, 278, 279, 280, 281, 282, 283, 284, 285, 286, 287, 288, 289, 290, 291, 292, 293, 294, 295, 296, 297, 298, 299, 300, 301, 302, 303, 304, 305, 306, 307, 308, 309, 310, 311, 312, 313, 314, 315, 316, 317, 318, 319, 320, 321, 322, 323, 324, 325, 326, 327, 328, 329, 330, 331, 332, 333, 334, 335, 336, 337, 338, 339, 340, 341, 342, 343, 344, 345, 346, 347, 348, 349, 350, 351].

Long non-coding *DACH1* worsens diabetic cardiomyopathy by increasing mitochondrial oxidative stress and promoting cell apoptosis through the degradation of SIRT3 [352]. In addition to histone modifications, miRNAs play a significant role in CH. The overexpression of miR-23a, miR-23b, miR-24, miR-195, or miR-214 has been shown to promote CH in neonatal cardiomyocytes, whereas the overexpression of miR-133 has the opposite effect, inhibiting the hypertrophic phenotype [353]. Moreover, inhibiting miR-143 causes the release of retinoic acid signaling from its repressed state, resulting in structural abnormalities in the outflow tracts and ventricles.

This phenomenon underscores an epigenetic link between the heartbeat and cardiac development, with miR-143 identified as a key player in the mechanotransduction process [354]. Families of miR-1 (which includes miR-1-1, miR-1-2, and miR-206) and miR-133 (comprising miR-133a-1, miR-133a-2, and miR-133b) are characterized by significant conservation. These families are primarily found in the heart, yet their expression is not confined solely to this tissue. The targeted removal of the muscle-specific miRNA, miR-1-2, has demonstrated multiple functions in cardiac physiology, including the regulation of heart morphogenesis, electrical conduction pathways, and cell cycle management [355].

The use of catheter-based delivery for anti-miR-21 has proven effective in inhibiting miR-21 in a porcine HF model, resulting in decreased cardiac fibrosis and hypertrophy, as well as improved heart function. In-depth RNA sequencing revealed that the suppression of miR-21 reduced inflammation and influenced various signaling pathways, with a notable decrease in the populations of macrophages and fibroblasts [356]. miRNAs are capable of either enhancing or inhibiting the death of cardiomyocytes and are involved in regulating neovascularization after ischemia [357]. Furthermore, these molecules can impact cardiac regeneration by controlling the proliferation of cardiomyocytes or by interfering with the protective effects provided by stem or progenitor cells. The role of miR-340-5p in the development of diabetic cardiomyopathy is critical, as it targets *Mcl-1*, leading to mitochondrial dysfunction, increased oxidative stress, and the apoptosis of cardiomyocytes [358]. The microRNA associated with cardiac fibrosis is miR-29, which exhibits decreased expression under conditions of cardiac stress. This reduction may lead to an increase in the production of extracellular matrix components by derepressing genes that encode collagens, elastin, or fibrillin [359].

Increasing evidence suggests that alterations in miR expression are associated with CVDs. Indeed, several microRNAs have been identified as promising therapeutic targets. For instance, the inhibition of miR-15 was shown to enhance cardiac function in animal models of HF by decreasing infarct size, fibrosis, and remodeling [360]. In neonatal mice, the suppression of the miR-15 family through locked nucleic acid-modified anti-miRNAs led to an elevated number of mitotic cardiomyocytes and the activation of CHEK1 [361]. The modulation of cardiac microRNAs has led to the validation of miR-208 as a powerful therapeutic target aimed at improving cardiac function and remodeling throughout the progression of heart disease. The increase in myocardial miR-208a was negatively linked to clinical outcomes and emerged as a significant independent predictor of cardiac death in a subsequent study [362]. Mice with a deletion in the miR-17/92 cluster experience death shortly after birth, which is attributed to hypoplastic lung development and heart defects caused by the concurrent loss of the miR-17, miR-18, and miR-92 seed families [363]. miR-497-5p intensifies dysfunction of endothelial cells resulting from oxidized low-density lipoprotein, which plays a pivotal role in the development of atherosclerosis. By targeting VEGFA and activating the p38/MAPK pathway, miR-497-5p fosters inflammation, oxidative stress, and cell mortality in endothelial cells [364]. MiR-126 exhibits the capability to promote vascular protection and mitigate the risk of atherosclerosis; the expression of miR-126 is stimulated by VEGF [365]. miR-30 was significantly lower during the early phase of a cardiac hypertrophic animal model and in human hearts that are failing. In contrast, both miR-214 and miR-30 showed increased levels in the maladaptive diseased heart, which adversely affects the expression of cardiac XBP1 and VEGF [366]. Meanwhile, circulating levels of miR-126 are markedly lower in patients with CAD, which is likely attributable to the inadequate packaging of miR-126 into endothelial microvesicles [365]. The increased levels of cyclin D2 reduced the effectiveness of miR-98 in counteracting Ang II-induced CH, highlighting that the antihypertrophic mechanisms of miR-98 are partially dependent on the downregulation of cyclin D2 [367].

miR-21_3p is identified as a significant paracrine RNA molecule that causes hypertrophy in cardiomyocytes. The targets of miR-21 include SORBS2 and PDLIM5. Thus, silencing these proteins in cardiomyocytes induced hypertrophic responses [368]. Interestingly, the long non-coding RNA Ahit is identified as a key regulator of CH, illustrating its role in preventing hypertrophy through the recruitment of the PRC2 protein SUZ12, which trimethylates the critical transcription factor MEF2A. Furthermore, the elevated levels of Ahit in patients suffering from hypertensive heart disease suggest its potential as a therapeutic target for CH [369]. MicroRNAs may represent new biomarkers for HF with preserved ejection fraction, diastolic dysfunction, and acute HF.

In rats with congestive HF, short-term vagus nerve stimulation reduced apoptosis through the downregulation of miR-205 [370]. The deletion of miR-1-2 in a homozygous state caused mortality during embryonic or perinatal stages, attributed to cardiac defects such as VSD, HF, and arrhythmias [371]. miR-1 expression acts to inhibit the WNT and FGF pathways by repressing *FZD7* and *FRS2*, respectively, thereby promoting the differentiation of cardiomyocytes and suppressing the development of endothelial cells. A study found that in patients with VSD, increased levels of GJA1 and SOX9 corresponded with a decrease in miR-1-1 expression, while elevated miR-181c levels were associated with reduced BMPR2 expression [372].

As a constituent of the miR-23/24/27 cluster, miR-24 has been reported to exhibit cardiomyocyte-protective effects *in vitro* [373]. Furthermore, *in vivo* studies indicate that the overexpression of miR-24 can mitigate infarct size and enhance cardiac function post-acute myocardial infarction. However, it is significant to note that cardiomyocyte-specific overexpression of miR-24 has been linked to embryonic lethality in murine models [373]. This research investigated circulating miRNAs (c-miRNAs) in children diagnosed with HF, comparing their levels before and after the implantation of a ventricular assist device [374]. The study identified six c-miRNAs that are integral to hemostatic regulation, with c-miR-409-3p being particularly significant as it is linked to a prothrombotic state through the downregulation of coagulation factors F7 and F2 [374]. Meanwhile, circSamd4, which is localized in the mitochondria, plays a significant role in cardiac regeneration by alleviating mitochondrial oxidative stress and minimizing DNA damage, whereby increased levels of circSamd4 promote the proliferation of cardiomyocytes, avert apoptosis, and enhance cardiac function post-MI [375].

The microRNAs hsa-let-7a, hsa-let-7b, and hsa-miR-486 were found to be significantly elevated in children with atrial septal defects. The overexpression of hsa-let-7a and hsa-let-7b was particularly noted in this group, as opposed to children with different subtypes of septal defects. Furthermore, a similar expression profile for hsa-let-7a and hsa-let-7b was observed in the mothers of children diagnosed with atrial septal defects [376]. A mouse model study conducted by researchers indicated that higher levels of miR-187 in endothelial cells are directly responsible for inducing CHD, particularly characterized by heart septal defects and a diminished heart size. miR-187 is directed towards *NIPBL*, a vital protein that plays a key role in attracting the cohesin complex and managing chromatin accessibility [377]. Mice that lack either miR-133a-1 or miR-133a-2 are typically normal, but the deletion of both miRNAs results in lethal VSD in approximately 50% of double-mutant embryos or neonates. Survivors who reach adulthood are likely to develop dilated cardiomyopathy and HF via the roles of miR-133a-1 and miR-133a-2 in cardiac development, gene expression, and function, suggesting that these miRNAs are integral to an SRF-dependent myogenic transcriptional pathway [222].

miR-222 was found to be upregulated in CH associated with exercise, and the implementation of cardiomyocyte-specific transgenic miR-222 offers a protective mechanism against cardiac remodeling resulting from I/R [378]. During cardiomyocyte hypertrophy, miRNAs undergo dynamic regulation, and the attenuation of miR-22 in rat cardiomyocytes effectively mitigates hypertrophic effects by alleviating the repression of PTEN [379]. In cases of pathological CH, the overexpression of miR-29a contributes to the progression of CH by regulating the PTEN/AKT/mTOR pathway and diminishing autophagy [221].

miR-21-3p is implicated in the regulation of HDAC8 expression and the AKT/Gsk3β pathway, indicating that therapeutic strategies focused on modulating miR-21-3p levels could be effective in combating CH [220]. The presence of miR-217 resulted in lower nitric oxide production, advanced endothelial dysfunction, increased blood pressure, and exacerbated atherosclerosis in mice prone to atherogenic conditions [380]. *MHRT* increases *KLF4* expression by either directly interacting with miR-145a-5p or by forming a complex with KLF4 that prevents its phosphorylation. This action inhibits the binding of ERK to KLF4, which subsequently reduces myocardin expression and lessens myocardial hypertrophy [381].

A dynamic imbalance between DNMT1 and TET2 is related to the hypermethylation of the miR-145 promoter. The reduction in miR-145 expression leads to the activation of NLRP3 inflammasome via the CD137/NFATc1 signaling cascade. miR-145 expression in plaques was subject to regulation through promoter hypermethylation, which was mediated by either DNMT1 or TET2 [382]. Inhibition of microRNA-210 through LNA-anti-microRNA-210 considerably advanced the differentiation process of Sca-1^+^ cardiac progenitor cells into cardiomyocytes [383]. A correlation exists between reduced miR-10a and increased GATA6/VCAM-1 in the cardiovascular endothelium, which is linked to the formation of atherosclerotic lesions in humans [384]. The deletion of *MEX3A* or *ATG5 in vivo* diminished the nuclear transport of miR-126-5p, heightened endothelial apoptosis, and intensified the progression of atherosclerosis [385].

A study involving a cohort of 820 participants over a decade revealed a notable correlation between three specific miRNAs (miR-126, miR-197, and miR-223) and the likelihood of experiencing MI, with adjustments made for potential confounding variables [386]. miR-449 plays a crucial role in regulating cTnI expression and enhancing cardiac function by inhibiting histone deacetylation mediated by HDAC1 at the *cTnI* promoter. This inhibition leads to increased histone acetylation (H3K4 and H3K9), which supports GATA4 binding and the transcriptional activation of cTnI in the hearts of older individuals [387]. The microRNAs miR-132, miR-140, and miR-210 were similarly linked to cardiovascular mortality in a cohort of 1112 patients [388]. Meanwhile, 28 miRNAs were found to be differentially expressed in diseased hearts, regardless of left ventricular assist device (LVAD) support [389]. Remarkably, the expression levels of 20 of these miRNAs showed normalization or reversal in the CHF group after receiving LVAD support, suggesting that these miRNAs may serve as valuable prognostic markers for patients suffering from end-stage CHF [389].

Subsequent experiments validated that H19 plays a role in regulating KDM3A expression, which helps to mitigate myocardial injury resulting from MI in a manner reliant on miR-22-3p [390]. The process of pri-miR221/222 maturation is positively modulated by METTL3 in an m6A-dependent manner, leading to the activation of WNT/β-catenin signaling through the inhibition of *DKK2*, which promotes CH induced by Ang II [391]. *TUG1* sequestered miR-132-3p, resulting in the increased expression of HDAC3. This upregulation reduced H3K9 acetylation and epigenetically repressed the expression of antioxidative genes such as *HSP70*, *BCL-XL*, and *PRDX2*, which are involved in the pathogenic development of AMI [392]. The suppression of miR-34 expression *in vivo* through the application of LNA-based anti-miRs or antagomiRs enhanced the survival of cardiomyocytes following AMI, consequently maintaining cardiac contractile function [388, 393]. Additionally, the inhibition of miR-34a contributed to improved cardiac performance in cases of moderate hypertrophic cardiomyopathy [393]. The function of miR-1 in myotonic dystrophy type 1 has been examined, revealing that a decrease in miR-1 levels is associated with the development of DCM. This study highlights Mp/Col15A1 as a significant target of miR-1, with its increased expression playing a role in the progression of DCM in myotonic dystrophy type 1 [394].

## 6. Epigenetic Drugs (Epi-Drugs) in Heart Diseases

**Fig. 7.**
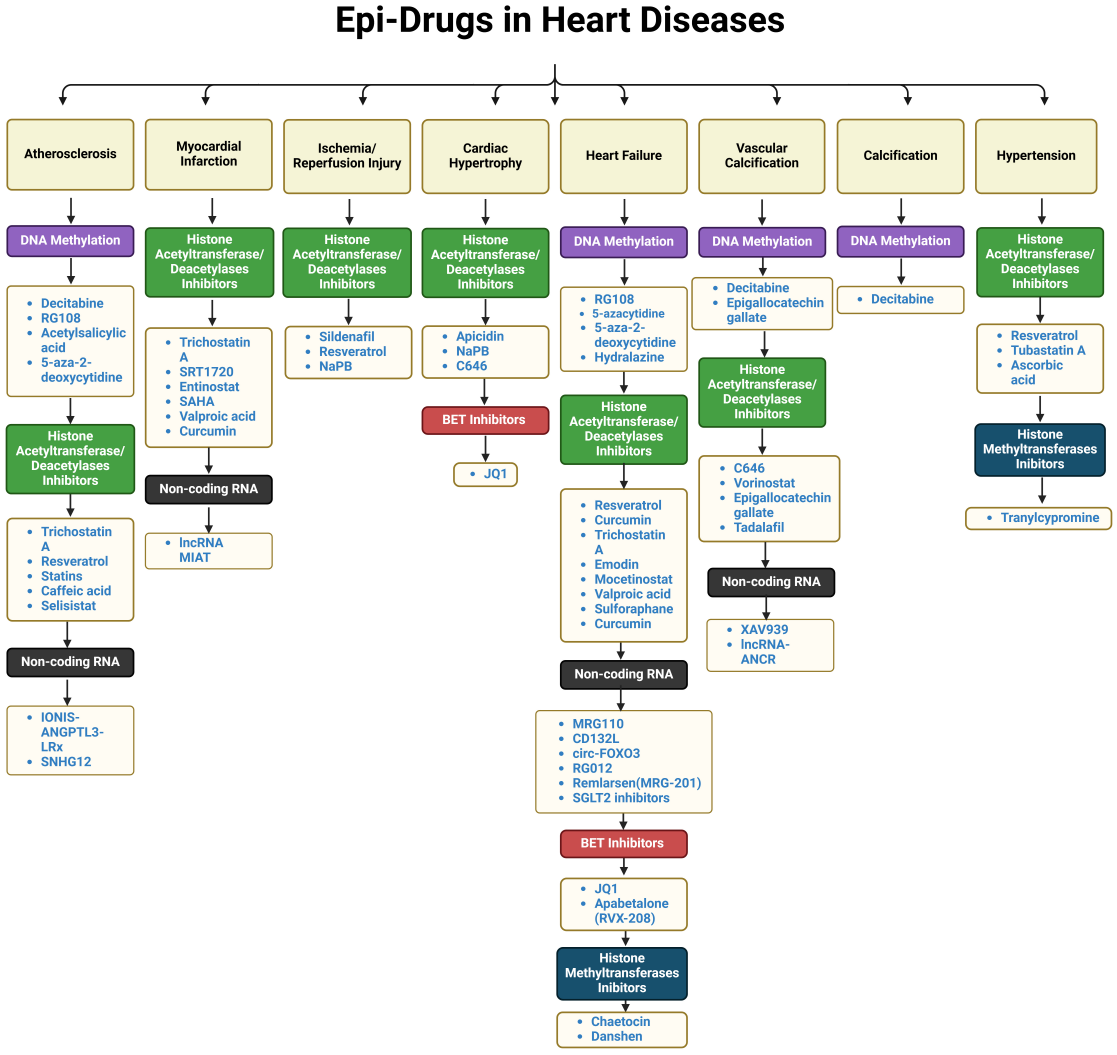
**Potential epigenetic-related drugs in heart diseases**. SNHG12, Small Nucleolar RNA Host Gene 12; SAHA, Suberoylanilide Hydroxamic Acid; NaPB, Sodium Phenylbutyrate; MIAT, Myocardial Infarction–Associated Transcript; BET, Bromodomain and Extra-Terminal; SGLT2, Sodium-Glucose Cotransporter 2; circ-FOXO3, Circular Forkhead box O3.

Various drug candidates that target epigenetic molecules have been discovered for cancer treatment and other diseases (Fig. 7). Additionally, some of these candidates have been used in research on CH and HF, involving both cellular and animal models.

It has been clinically established that trimetazidine and metabolic-related substances, including levocarnitine, coenzyme Q10, and phosphocreatine, possess impressive cardioprotective capabilities by regulating changes in cardiac metabolism [395, 396, 397]. Chronic pericarditis is identified as the predominant cardiotoxic effect of radiotherapy. HF is the major cardiotoxicity associated with anthracycline drugs, occurring at an incidence rate of about 5% to 23% [398]. Dexrazoxane is recognized as the only cardioprotective drug approved by the FDA for the treatment of cardiotoxicity induced by anthracycline agents [399]. Chaetocin treatment was found to lower the expression of H3K9me3, reduce the formation of atherosclerotic plaques, and enhance the stability of these plaques. This was achieved by reducing the area of the necrotic core and lipid deposits, while increasing collagen levels and promoting a contractile phenotype in vascular smooth muscle cells [400].

The administration of atorvastatin led to a reduction in the expression of miR-34a and an elevation in SIRT1 levels; conversely, rosuvastatin, a different class of statin, did not produce similar outcomes [401]. Meanwhile, the HDAC inhibitor SAHA was shown to improve cardiac structure and function in a feline model of pressure overload-induced HFpEF. The administration of SAHA reduced left ventricular hypertrophy and diastolic dysfunction, possibly through its favorable effects on mitochondrial metabolism [402]. The pan-HDAC inhibitor SAHA, which also acts as a class I and IIb HDAC inhibitor, successfully elevated miR-133a levels in mice that experienced TAC, reducing CTGF, collagen, and fibrosis within the myocardium [403]. Apagliflozin and empagliflozin, both SGLT2 inhibitors, have demonstrated a significant decrease in all-cause and cardiovascular mortality, as well as in hospitalizations for HF and renal events in individuals with HF with reduced ejection fraction (HFrEF), independent of their diabetes status. The benefits were consistently evident across various subgroups, underscoring the significance of SGLT2 inhibitors in managing HFrEF [404].

The phase 1b clinical trial investigated the safety and impact of CDR132L, a miR-132 inhibitor, in patients with chronic ischemic HF. The study found that CDR132L was safe and well-tolerated, promoting a dose-dependent reduction in miR-132 levels. Patients who received doses of ≥1 mg/kg displayed enhancements in heart function, including lower NT-proBNP levels and QRS narrowing [405]. The synthetic miR-132 inhibitor CDR132L was safely and effectively administered on a monthly basis in a pig model of chronic CHF after MI. This treatment not only improved systolic and diastolic cardiac function but also reversed pathological remodeling [406].

The simultaneous upregulation of miR-129-5p and the reduction in CDK6 expression were mechanistically involved in the trichostatin A (TSA)-mediated inhibition of H9c2 cell proliferation [407]. G9a, a histone methyltransferase, is crucial in the development of HF following AMI. Additionally, the therapeutic combination of erythropoietin and a G9a inhibitor has demonstrated efficacy in preserving both the architecture and functional integrity of the left ventricle in AMI-induced rat models [408]. The remarkable activation of SIRT1/Nrf2 signaling is primarily linked to the effects of bakuchiol, which ultimately fortified the antioxidative capacity of the heart by boosting antioxidant production and reducing the formation of reactive oxygen species [409].

Multiple classes of HDAC inhibitors, such as TSA, vorinostat, hydroxamic acid, and sodium butyrate, are known to reduce the expression and activity of HDACs, thereby shifting the overall balance toward enhanced histone acetylation [410, 411]. The anti-arrhythmic effects of TSA seem to be unaffected by the expression of Ang II, TGF-β1, ERK1/2, shifts in atrial refractoriness, or variations in intracardiac pressure [412]. Silencing miR-541 can obstruct the antihypertrophic effects associated with MITF knockdown in cardiomyocytes subjected to Ang II treatment. The influence of MITF on CH is dependent on the regulation of miR-541 [234]. miR-19b was found to significantly elevate pro-hypertrophic calcineurin/NFAT signaling. The genes *atrogin-1* and *MuRF-1*, which are involved in anti-hypertrophic processes, are directly influenced by miR-19a/b. Furthermore, the application of the calcineurin inhibitor cyclosporin A and protein kinase C inhibitor GF10923X effectively diminished the miR-19b-induced increase in cell size and the expression of hypertrophic markers [413].

Numerous cardiovascular disorders are identified by the abnormal methylation of CpG islands, resulting in the persistent investigation of targeted drugs that could inhibit DNA methyltransferase, either directly or by reducing its gene expression, including hydralazine and procainamide [414]. The cause of the toxicity was identified as procainamide. Moreover, as procainamide-related lung fibrosis is an uncommon condition, this case is shared to highlight the potential risks associated with procainamide and is imperative for diligent monitoring in patients who have undergone surgery [415].

Apabetalone (APA) significantly lowered the incidence of major adverse cardiovascular events and HF hospitalizations in patients with chronic kidney disease and type 2 diabetes after acute coronary syndrome. These results imply that apabetalone may deliver cardiovascular protection to this high-risk population by modulating epigenetic factors [416]. APA, which acts as a BET protein inhibitor, aids in the restoration of angiogenesis in diabetic conditions by epigenetically downregulating the antiangiogenic gene *THBS1* while preserving VEGFA signaling pathways. In diabetic mice with limb ischemia, treatment with APA resulted in enhanced vascularization and perfusion [417]. The binding of APA to BRD4 may influence cholesterol levels and inflammation; specifically, APA promotes the expression of the *ApoA-I* gene through the mediation of BET family member BRD4. APA may play a role in reducing CH and fibrosis, indicating promising new options for HF treatment [418]. JQ1-mediated inhibition of BET bromodomains suppresses proinflammatory gene expression in cardiac fibroblasts, thereby reducing fibrosis, preventing adverse remodeling, and improving survival in a mouse model of DCM. The identification of BRD4 as a significant regulator of NF-κB-driven inflammation emphasizes the promise of BET inhibition as a therapeutic strategy for chronic DCM [419]. The mechanisms through which azacitidine exerts its antineoplastic effects are thought to include two main processes: cytotoxicity, stemming from its incorporation into RNA and DNA, and DNA hypomethylation, which restores normal growth control and differentiation in hematopoietic cells [48]. Azacitidine functions by stoichiometrically binding to DNMT1, thereby preventing the methylation of replicating DNA and inducing a hypomethylated state in the DNA. The occurrence of pressure overload triggers pathological remodeling of the heart, linked to an increase in DNA methylation. Low-dose treatment with 5-azacytidine lowers this methylation and alleviates both hypertrophy and fibrosis [420].

The mechanism through which zebularine functions as a DNA methylation inhibitor is thought to involve its integration into DNA, a process that presumably follows the phosphorylation of zebularine to the diphosphate level and its conversion into a deoxynucleotide. Zebularine is classified as a stable agent for DNA demethylation and is the first drug in its class capable of reactivating an epigenetically silenced gene through oral delivery [421].

RG108 functions as an inhibitor of DNA methyltransferase, characterized by its unique properties that will be especially beneficial for the experimental manipulation of epigenetic gene regulation [422]. C646, a p300 inhibitor, improves coronary flow reserve, cardiac function, and vascular health in SIRT3 knockout mice. The mechanism of action for C646 involves reducing p300 and H3K56 acetylation, leading to enhanced endothelial function and the suppression of inflammation-related pathways, including NF-κB [423]. Curcumin, among other HAT inhibitors, inhibits the acetylation of histones H3 and H4 by p300 and CBP. This mechanism leads to a decrease in cell proliferation and has been researched for its potential role in preventing HF in rat subjects [424]. Curcumin was also found to decrease myocardial dysfunction, oxidative stress, and apoptosis in the cardiac tissue of diabetic rats. The administration of curcumin increased phosphorylation of *AKT* and decreased acetylation of *FOXO1*, thereby playing a crucial role in the management of DCM by modulating the Sirt1–Foxo1 and PI3K–AKT pathways [425].

A significant decrease in histone 3 acetylation has been detected in cells treated with doxorubicin (DOX). The treatment of cultured cardiomyocyte precursor cells with DOX induced severe apoptosis-related cell death, which correlates with heightened oxidative stress [426]. The administration of DOX can also elevate the activity of demethylases KDM3A and JMJD3, resulting in higher levels of histone methylation [427]. The negative regulation of *SESN2* by JMJD3 occurs through a decrease in H3K27me3 enrichment at the *SESN2* promoter region, leading to mitochondrial dysfunction and cardiomyocyte apoptosis. Therefore, targeting the JMJD3–SESN2 signaling axis could be a promising therapeutic strategy for preventing DOX-induced cardiomyopathy [428, 429]. The analysis of microRNAs in the hearts of individual mice revealed that miR-34a was significantly elevated post-DOX treatment; meanwhile, miR-205 experienced a significant decline after the combined treatment of imatinib mesylate and DOX [430].

The hyperacetylation mimic of GATA4 maintains its protective effect against DOX, while the hypoacetylation mimic is unable to sustain this protective capacity. The SIRT6–TIP60–GATA4 axis is essential for promoting the anti-apoptotic pathway, which contributes to the reduction of DOX-associated toxicity [431]. DOX induces various regulated pathways of cardiomyocyte death, which include autophagy, ferroptosis, necroptosis, pyroptosis, and apoptosis [432]. The administration of DOX leads to a decrease in the levels of NAD^+^-dependent histone deacetylases SIRT1, SIRT3, and SIRT6, and an increase in the levels of Zn^2+^-dependent histone deacetylase HDAC1, which together mediate changes in histone acetylation modifications [433]. Betulin provided substantial protection against diabetic cardiomyopathy, which was associated with the modulation of the Siti1/NLRP3/NF-κB signaling pathway [434]. The protective effects of miR-152 against DOX-induced cardiac injury in mice were compromised due to a deficiency in *Nrf2* [432]. The administration of dapagliflozin in diabetic mice resulted in the alleviation of Ang II-induced cardiomyopathy, characterized by reductions in inflammation, fibrosis, and intracellular calcium levels [435]. The expression of DNMT1 was found to be elevated in the cardiac tissues of rats subjected to DOX treatment, exhibiting an inverse relationship with the expression levels of miR-152-3p. It was demonstrated that DNMT1 promotes the methylation of the promoter region of miR-152-3p, thereby reducing its expression and resulting in the inhibition of mitophagy in H9c2 cells. Furthermore, the depletion of DNMT1 conferred protection against HF in rats through a mechanism dependent on miR-152-3p, ETS1, and RhoH [436]. Sulforaphane defends the heart against Ang II-induced harm by epigenetically activating Nrf2. Moreover, sulforaphane lowers DNA methylation levels and raises histone acetylation in the *Nrf2* promoter, thereby enhancing antioxidant defenses and contributing to sustained cardioprotection [437].

Antiretroviral medications influence the epigenetic alterations of cardiac cells by interacting with histone markers that are essential for gene expression. Indeed, antiretroviral medications are particularly significant in the process of histone deacetylation at the H3K9 and H3K27 sites during periods of cellular stress [438]. Allisartan isoproxil has been shown to alleviate DCM by reducing oxidative stress and inflammation associated with diabetes through the SIRT1/Nrf2/NF-κB signaling pathway [439]. Hop functions to inhibit SRF-dependent transcriptional activation through the recruitment of HDAC activity and the formation of a complex that contains HDAC2. Transgenic mice that overexpress Hop are prone to severe CH, cardiac fibrosis, and early mortality when subjected to a two-week treatment with the hydroxamic acid pan-HDAC inhibitor, TSA, or the short-chain fatty acid, valproic acid [440].

The apicidin derivative, referred to as API-D, is effective in decreasing hypertrophy and, consequently, in preventing the transition to HF in mice subjected to TAC [441]. Elabela treatment was observed to have significant protective effects against oxidative stress in the heart due to diabetes, potentially relying heavily on the deacetylation of *FOXO3A* mediated by SIRT3 [442]. The effects of scriptaid did not entirely remove hypertrophic growth, implying that HDAC-dependent pathways do not control certain aspects of the growth response. A dose-dependent decrease in hypertrophy was recorded following the use of scriptaid [443]. As a natural H_2_S donor, erucin protected vascular cells against damage induced by high glucose concentrations by lowering oxidative stress, inflammation, and endothelial permeability [444]. The downregulation of the GRIN2D-mediated calcium pathway by miR-129-1-3p provides a protective mechanism against apoptosis in cardiomyocytes triggered by pirarubicin [445].

## 7. Conclusions

Heart diseases remain the leading factor in worldwide fatalities, which accentuates the urgent demand for improved insights into their multifaceted etiology. Epigenetic modifications, which encompass DNA methylation, histone changes, and non-coding RNA activity, contrast with traditional genetic mutations by impacting gene expression without altering the DNA sequence. This review illustrates the fundamental impact of epigenetic mechanisms, which encompass DNA methylation, histone modifications, and ncRNAs, on the onset and evolution of several CVDs. These mechanisms influence cellular responses to environmental factors, developmental prompts, and pathological stress, and are connected to heart diseases. Tracing back to early insights in epigenetics and advancing to the present-day classification of ‘writers (e.g., DNMTs, HATs, and methyltransferases), erasers (e.g., TET proteins, HDACs, and demethylases), and readers (e.g., BRD4, BRG1, MeCP2)’, the regulation of gene expression through reversible, non-genetic changes has been recognized as a vital contributor to heart development, functionality, and pathology. Dysfunctions in these proteins have been closely correlated with HF, CAD, and congenital heart defects. Enzymes, particularly DNMTs, HDACs, SIRTs, and TETs, are notably important as regulators with substantial potential for diagnostic and therapeutic applications. The sophisticated interplay of these components governs vital processes including CH, fibrosis, and apoptosis. Additionally, specific microRNAs and long non-coding RNAs have been identified as viable biomarkers and therapeutic targets, playing a role in various processes, including HF and CH. The therapeutic potential of miRNA-based interventions, including antagomiRs and mimic therapies, is receiving growing interest. This review highlights the role of ncRNAs as both biomarkers and targets in the development of personalized epigenetic therapies. The application of epigenetic drugs, some of which are presently in clinical trials, paves the way for innovative approaches in precision cardiology. Epigenetic variations are the source of changes in gene expression that play a role in the development and maintenance of fatal diseases such as heart disease. Increasing interest in epigenetic research is focusing on pharmacological interventions that epigenetically reverse the characteristics of heart disease. In DNMT and HDAC inhibition, epigenetic modifiers act as agents or can be combined with various established therapies. Therefore, combination therapies with epigenetic modifiers that enhance both the adjuvancy and antigenicity of heart disease treatments are promising approaches against heart disease.

Ongoing research that combines next-generation sequencing, machine learning, and patient-specific epigenetic profiling will be imperative for applying these discoveries to personalized therapies. The convergence of epigenomics alongside other omics technologies, such as transcriptomics and proteomics, is essential for elucidating the complex regulatory systems implicated in heart disease. Exploring and adjusting the epigenetic landscape represents a significant opportunity to reverse maladaptive cardiac remodeling and enhance cardiovascular health outcomes globally. The ability to understand and manipulate the epigenome has the potential to fundamentally change the approach to heart disease prevention.
